# Glyceraldehyde‐3‐phosphate dehydrogenase from *Citrobacter* sp. S‐77 is post‐translationally modified by CoA (protein CoAlation) under oxidative stress

**DOI:** 10.1002/2211-5463.12542

**Published:** 2018-11-28

**Authors:** Kohsei Tsuji, Ki‐Seok Yoon, Seiji Ogo

**Affiliations:** ^1^ Centre for Small Molecule Energy Kyushu University Fukuoka Japan; ^2^ Department of Chemistry and Biochemistry Graduate School of Engineering Kyushu University Fukuoka Japan; ^3^ International Institute for Carbon‐Neutral Energy Research (WPI‐I2CNER) Kyushu University Fukuoka Japan

**Keywords:** coenzyme A, glyceraldehyde‐3‐phosphate dehydrogenase, post‐translational modification, redox regulation, S‐thiolation

## Abstract

Protein CoAlation (S‐thiolation by coenzyme A) has recently emerged as an alternative redox‐regulated post‐translational modification by which protein thiols are covalently modified with coenzyme A (CoA). However, little is known about the role and mechanism of this post‐translational modification. In the present study, we investigated CoAlation of glyceraldehyde‐3‐phosphate dehydrogenase (GAPDH) from a facultative anaerobic Gram‐negative bacterium *Citrobacter* sp. S‐77 (*Cb* GAPDH). GAPDH is a key glycolytic enzyme whose activity relies on the thiol‐based redox‐regulated post‐translational modifications of active‐site cysteine. LC‐MS/MS analysis revealed that CoAlation of *Cb* GAPDH occurred *in vivo* under sodium hypochlorite (NaOCl) stress. The purified *Cb* GAPDH was highly sensitive to overoxidation by H_2_O_2_ and NaOCl, which resulted in irreversible enzyme inactivation. By contrast, treatment with coenzyme A disulphide (CoASSCoA) or H_2_O_2_/NaOCl in the presence of CoA led to CoAlation and inactivation of the enzyme; activity could be recovered after incubation with dithiothreitol, glutathione and CoA. CoAlation of the enzyme *in vitro* was confirmed by mass spectrometry. The presence of a substrate, glyceraldehyde‐3‐phosphate, fully protected *Cb* GAPDH from inactivation by CoAlation, suggesting that the inactivation is due to the formation of mixed disulphides between CoA and the active‐site cysteine Cys149. A molecular docking study also supported the formation of mixed disulphide without steric constraints. These observations suggest that CoAlation is an alternative mechanism to protect the redox‐sensitive thiol (Cys149) of *Cb* GAPDH against irreversible oxidation, thereby regulating enzyme activity under oxidative stress.

**Abbreviations**
CAM: carbamidomethylation
CID: collision‐induced dissociation
CoAlation: S‐thiolation by coenzyme A
CoA: reduced coenzyme A
CoASSCoA: coenzyme A disulphide
DDA: data‐dependent acquisition
DTT: dithiothreitol
G3P: glyceraldehyde‐3‐phosphate
GAPDH: glyceraldehyde‐3‐phosphate dehydrogenase
Grx: glutaredoxin
GSH: reduced glutathione
GSSG: glutathione disulphide
IAM: iodoacetamide
LC‐MS/MS: liquid chromatography tandem mass spectrometry
MALDI‐TOF‐MS: matrix‐assisted laser desorption ionisation–time‐of‐flight mass spectrometry
NaOCl: sodium hypochlorite
SDS/PAGE: sodium dodecyl sulfate/polyacrylamide gel electrophoresis
Trx: thioredoxin

Protein S‐thiolation is an important redox post‐translational modification that involves formation of mixed disulphides between the redox‐sensitive cysteines of proteins and cellular low‐molecular‐weight thiols, such as glutathione (GSH), cysteine (Cys), bacillithiol, mycothiol and coenzyme A (CoA) 1, 2, 3, 4, 5, 6. This modification can protect protein thiols against irreversible oxidation and functions via a redox‐controlled mechanism to regulate the activity and functions of thiol proteins in response to oxidative and nitrosative stresses 2, 4. Because GSH represents the most abundant cellular redox buffer (1–10 mm) in most eukaryotic and prokaryotic cells 4, 5, the physiological role and importance in redox regulation of S‐glutathionylation has been extensively studied over the last several decades 7. However, the presence of other low‐molecular‐weight thiols in the cell suggests that alternative S‐thiolation is also possible; for example, S‐cysteinylation in *Salmonella* *typhimurium* 8, S‐thiolation by glutathionylspermidine in *Escherichia coli* 9, S‐bacillithiolation in Firmicutes 10 and S‐mycothiolation in Actinomycetes 11. In contrast to S‐glutathionylation, little is known about other forms of S‐thiolation by cellular low‐molecular‐weight thiols, particularly CoA that might alternatively contribute to redox regulation.

CoA is a cellular low‐molecular‐weight thiol that participates in > 100 reactions of essential intermediary metabolisms and is required for approximately 4% of enzymatic reactions in the cell 12, 13. Aside from its metabolic importance, CoA has been known to form persulphide (CoASSH), mixed disulphides with GSH, Cys (CoASSG, CoASSCys) and even protein thiols (PSSCoA) endogenously, which indicated the biological relevance of CoA‐based redox regulation 5, 14, 15, 16, 17. An early study showed that approximately 45% of CoA was found to have a disulphide linkage to protein in spore‐forming *Bacillus megaterium* 18. Later, some proteins have been identified as targets of covalent modification with CoA from biochemical and crystallographic studies, including RimL 19, acetyl‐CoA acetyltransferase 20, flavodoxin 21 and OhrR 22. Moreover, a recent study using an anti‐CoA monoclonal antibody and tandem mass spectrometry (MS) demonstrated that some key metabolic enzymes in mammalian and prokaryotic cells were reversibly modified by CoA under oxidative or metabolic stresses 6, 23. In this context, post‐translational modification that involves formation of mixed disulphides between CoA and protein thiols has been recently termed CoAlation (S‐thiolation by CoA) 6, 23. The contents of CoAlated proteins were altered in response to stresses, and CoAlation of the identified proteins reversibly changed their activities. However, the induction and reduction mechanisms of protein CoAlation remain unclear.

In the present study, we investigated CoAlation of glyceraldehyde‐3‐phosphate dehydrogenase (GAPDH; EC 1.2.1.12) from a facultative anaerobic Gram‐negative bacterium *Citrobacter* sp. S‐77 (*Cb*GAPDH). GAPDH is a key glycolytic enzyme that catalyses reversible phosphorylation of glyceraldehyde‐3‐phosphate (G3P) to 1,3‐bisphosphoglycerate (1,3‐BPG) in the presence of inorganic phosphate (P_i_) and NAD^+^ 4, 24. GAPDH is a multifunctional protein that has numerous functions unrelated to glycolysis such as DNA repair, cell death and stress response 25, 26. The protein is highly abundant in cells (consisting of 5–20% of cytosolic protein) 25 and has been identified as the most prominent target of S‐thiolation among various kinds of organisms on the basis of proteomic studies, including plant, mammalian, yeast and bacteria 6, 23, 27, 28, 29, 30, 31. GAPDH possesses a conserved active‐site cysteine (hereafter, Cys149), which binds the substrate by forming a thiohemiacetal intermediate during catalysis 24. The efficient catalytic reaction of the enzyme is facilitated by the p*K*_a_‐lowering microenvironment near the active site where the deprotonation of Cys149 by His176 makes it more acidic (p*K*_a_: 5.5–6.0) 25. Moreover, Cys149 is readily susceptible to oxidation to sulphenate (‐SO^−^) and subsequent S‐thiolation 27, 28, 32, 33, 34. Although the mechanism of S‐thiolation *in vivo* remains controversial, GAPDH is known to undergo S‐glutathionylation nonenzymatically by incubation with glutathione disulphide (GSSG), H_2_O_2_ plus GSH, or nitrosoglutathione (GSNO) *in vitro*, which results in reversible enzyme inactivation due to modification of catalytic cysteine 32, 33, 34, 35. S‐thiolated GAPDH homologs are enzymatically reactivated by glutaredoxin (Grx), thioredoxin (Trx) or its analogous systems, which are highly conserved across all living organisms 27, 28, 32, 33, 36, 37, 38. The consequence of S‐thiolation of GAPDH is inhibition of its glycolytic activity and downregulation of glycolysis, which results in redirection to the pentose phosphate pathway for providing a reducing equivalent of NAPDH as a means of adaptation under oxidative stress 29, 39. Besides S‐glutathionylation, bacillithiol and mycothiol also participate in redox regulation of thiol proteins by protein S‐bacillithiolation and S‐mycothiolation in Gram‐positive pathogenic bacteria, such as *Staphylococcus aureus* and *Corynebacterium diphtheriae* under oxidative stress 27, 28. In both bacteria, GAPDH was identified as most abundant target of S‐thiolation under NaOCl stress and its glycolytic activity is redox‐regulated by S‐bacillithiolation and S‐mycothiolation of active‐site cysteine of GAPDH homologs. Furthermore, recent study showed that protein CoAlation is a widespread redox‐regulated post‐translational modification that occurs in Gram‐positive and negative bacteria under oxidative and metabolic stress 23. Of note, two GAPDH isoforms of *S. aureus* were identified as a target of protein CoAlation in the bacterium exposed to oxidative stress.

Here, we studied the CoAlation of *Cb*GAPDH under NaOCl stress. The peptide containing active‐site Cys149 of *Cb*GAPDH was identified as a target of CoAlation in *Citrobacter* sp. S‐77 cell exposed to NaOCl *in vivo*. Further, redox regulation of *Cb*GAPDH via CoAlation was examined by *in vitro* and *in silico* approaches including biochemical assay, mass spectrometry, circular dichroism and molecular docking. CoAlation of *Cb*GAPDH resulted in reversible inactivation to protect vulnerable cysteine of the enzyme against irreversible oxidation, and the CoAlated enzyme was reactivated by dithiothreitol (DTT), GSH and CoA *in vitro*. Therefore, we showed that CoAlation is an alternative mechanism of thiol protection and redox regulation of *Cb*GAPDH under oxidative stress in a way analogous to S‐glutathionylation.

## Results

### *In vivo* CoAlation of *Cb*GAPDH under hypochlorite stress

**Figure 1:**
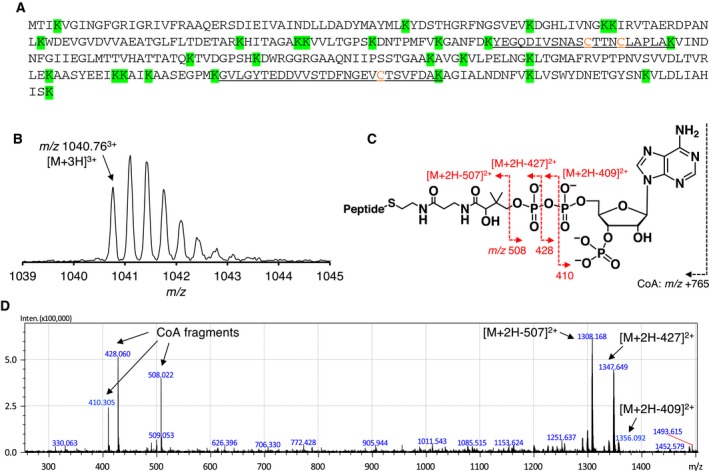
*In vivo* CoAlation of *Cb* GAPDH under NaOCl stress. (A) The predicted cleavage sites in the sequence of *Cb* GAPDH after Lys‐C digestion are depicted in green background, and the resulting peptides containing Cys149, Cys153 and Cys288 (Y138–K159 and G269–K295) are underlined with Cys residues coloured by orange character. (B) The precursor ion of CoAlated peptide (*m/z* 1040.76^3+^) (C) Schematic illustration of fragmentations of CoA by CID during MS/MS spectra acquisitions. Neutral loss of precursor ions characteristic to the loss of CoA fragments (*m/z* 410, 428, 508) by CID served for verification of the CoAlated peptides. (D) The MS/MS spectrum of *in vivo* CoAlated peptide of *Cb* GAPDH under NaOCl stress. The abundance of the peaks of precursor ions minus CoA fragments led to decreased intensity of y‐ and b‐ion series, which hampered identification of the peptide backbone fragmentations. The results presented here are one of the representatives of two independent experiments.

In the present study, we investigated whether *Cb*GAPDH could also be a target of CoAlation under NaOCl stress *in vivo*. The strain S‐77 was grown in M9 minimal medium to late‐logarithmic phase (OD_500_ = 1.0) and then exposed to 500 μm NaOCl for 30 min to induce oxidative stress. We next analysed *in vivo* redox modifications of *Cb*GAPDH in the bacteria with/without NaOCl stress by using LC‐MS/MS analysis after in‐gel digestion by Lys‐C. Prediction of cleavage site of *Cb*GAPDH suggested that two Cys containing peptide could be sliced after Lys‐C digestion, namely, a Cys149 and 153 containing peptide and a Cys288 containing peptide (Fig. 1A). We focused on these Cys containing peptides to examine the redox modifications. The list of identified Cys containing peptides of *Cb*GAPDH with/without NaOCl treatment is shown in Table 1. Under NaOCl stressed conditions, triple charged precursor ion (*m/z* 1040.76) that corresponded to a Cys149 and Cys153 containing peptide modified by one CoA and one carbamidomethylation (CAM) was detected (Fig. 1B). Interestingly, the MS/MS spectrum of Fig. 1D clearly showed the characteristic peaks of CoA fragments from the precursor ion as indicated by the loss of 410, 428 and 508 Da (Fig. 1C,D), as has been observed in the recently reported publication 6. The peptide peaks of corresponding precursor ions minus CoA fragments (−409, −427 and −507 Da) were also abundant in the MS/MS spectrum. However, we failed to identify the CoAlated cysteine presumably due to inefficient peptide backbone fragmentation and low abundance of the peptide. Instead, Cys153 and Cys288 were largely carbamidomethylated under both nonstressed and NaOCl stressed conditions. It should be noted that intramolecular disulphide bonding (Cys149‐S‐S‐Cys153) (*m/z* 1148.55) was also detected, which suggested that it might be an additional form of redox modification of *Cb*GAPDH. Overall, the results demonstrated that endogenous CoAlation of *Cb*GAPDH occurred in the active‐site Cys149 and Cys153 containing peptide under NaOCl stress *in vivo*.

**Table 1:** Identification of cysteine containing peptides of *Cb*GAPDH under nonstress and NaOCl stress conditions using LC‐MS/MS analysis. The *Citrobacter* sp. S‐77 grown to late‐logarithmic phase was exposed to 500 μm NaOCl for 30 min. Then, cytoplasmic protein extract was prepared in IAM containing lysis buffer, separated using nonreducing SDS/PAGE, digested with Lys‐C and analysed by LC‐MS/MS as described in Materials and methods

Sequence positions	Modified peptide sequences	Modified cysteines	Cysteine modifications	Theoretical peptide mass (*m/z*)	Observed precursor ion (*m/z*)	Charge (*z*)	Error (Da)
NaOCl stressed conditions							
Y138‐K159	YEGQDIVSNAS**C**_**149**_(+CoA/CAM)TTN**C**_**153**_(+CoA/CAM)LAPLAK	149, 153	CoAlation, CAM	1040.732	1040.765	3	0.033
Y138‐K159	YEGQDIVSNAS**C**_**149**_(–2)TTN**C**_**153**_LAPLAK	149, 153	Disulphide	1148.529	1148.556	2	0.026
Y138‐K159	YEGQDIVSNAS**C**_**149**_(+SO_3_H)TTN**C**_**153**_(+CAM)LAPLAK	149, 153	SO_3_H, CAM	1202.040	1202.076	2	0.036
Y138‐K159	YEGQDIVSNAS**C**_**149**_(+CAM)TTN**C**_**153**_(+CAM)LAPLAK	149, 153	CAM, CAM	1206.559	1206.580	2	0.021
G269‐K295	GVLGYTEDDVVSTDFNGEV**C**_**288**_(+CAM)TSVFDAK	288	CAM	1462.657	1462.677	2	0.020
Nonstressed conditions							
Y138‐K159	YEGQDIVSNAS**C**_**149**_(+CAM)TTN**C**_**153**_(+CAM)LAPLAK	149, 153	CAM, CAM	1206.559	1206.589	2	0.030
Y138‐K159	YEGQDIVSNAS**C**_**149**_(+CAM)TTN**C**_**153**_(+PA)LAPLAK	149, 153	CAM, PA	1213.566	1213.596	2	0.029
G269‐K295	GVLGYTEDDVVSTDFNGEV**C**_**288**_(+CAM)TSVFDAK	288	CAM	1462.657	1462.686	2	0.028

Cys149, Cys153 and Cys288 are indicated by bold character.

### Purification and biochemical characterisation of *Cb*GAPDH

Next, we were interested in the consequence of redox modification of *Cb*GAPDH by CoAlation, such as changes in enzyme activity and protein structure. We thus aimed to obtain purified *Cb*GAPDH to conduct precise *in vitro* study of *Cb*GAPDH CoAlation. GAPDH was purified to electrophoretic homogeneity from *Citrobacter* sp. S‐77 with a purification fold increase of 4.9, yield of 22% and specific activity of 145.6 U·mg^−1^ (Table S1). The molecular weight of native *Cb*GAPDH was determined to be approximately 140 kDa by gel filtration, and the band of purified enzyme appeared at 35 kDa on sodium dodecyl sulfate/polyacrylamide gel electrophoresis (SDS/PAGE) (Fig. S1), which indicated that native *Cb*GAPDH is a homotetramer. According to the draft genome sequence of the strain S‐77 (BioProject, ASM73967v1) 40, GAPDH‐like genes, *gapA* and *gapC*, with high similarity were identified. The N‐terminal amino acid sequence of purified GAPDH was ‘TIKVGINGFG’, which is identical to the amino acid sequence of GapA. We thus confirmed that purified *Cb*GAPDH was GapA. The amino acid sequence alignment was constructed on the basis of other sequences of GAPDHs (Fig. S2). *Cb*GAPDH was highly homologous to GAPDH from *E. coli* and *S*. Typhimurium (96%), but not very similar to that of mammalian (66–68%) or Gram‐positive bacteria (46–51%). A steady‐state kinetic analysis of *Cb*GAPDH showed that the *K*_m_ values for G3P, NAD^+^ and P_i_ were 1.23 ± 0.19, 0.12 ± 0.01 and 3.65 ± 0.34 mm, with a *k*_cat_ of approximately 360 s^−1^ (Table 2). These values are comparable with GAPDHs from *E*. *coli* and *Geobacillus stearothermophilus* 24, 41.

**Table 2:** Steady‐state kinetic parameters of *Cb*GAPDH. The kinetic parameters were calculated from at least five experiments with standard deviations

Substrate	*V*_max_ (U·mg^−1^)	*K*_m_ (mm)	*k*_cat_ (s^−1^)	*k*_cat_/*K*_m_ (M^−1^·s^−1^)
G3P	152 ± 7.5	1.23 ± 0.19	360 ± 18	2.9 × 10^5^
NAD^+^	152 ± 3.5	0.12 ± 0.01	360 ± 8.3	3.0 × 10^6^
P_i_	149 ± 4.0	3.65 ± 0.34	352 ± 9.4	9.6 × 10^4^

### Inactivation of *Cb*GAPDH by CoASSCoA

**Figure 2:**
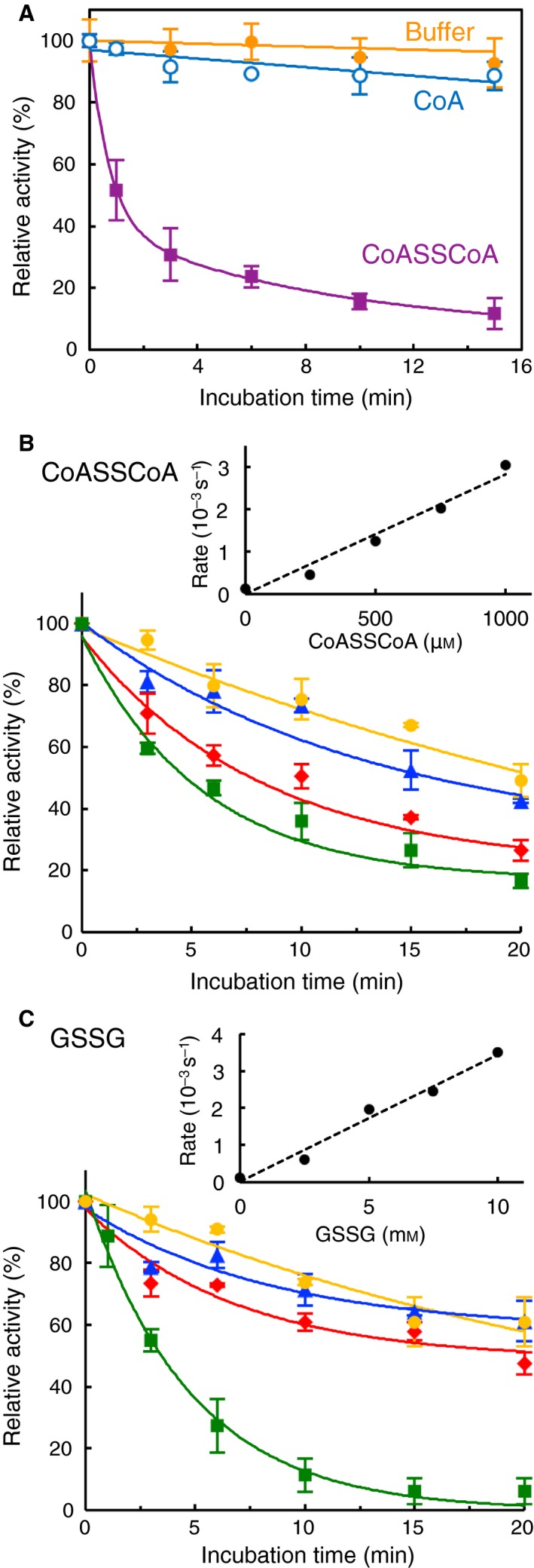
Inactivation profile of *Cb* GAPDH by CoASSCoA and GSSG. (A) Time‐course inactivation of *Cb* GAPDH by CoASSCoA. The enzyme was incubated with 1 mm CoASSCoA (■), 1 mm CoA (○) or buffer as control (◆) for 0–15 min. (B) Time‐dependent inactivation of *Cb* GAPDH by CoASSCoA, 250 μm (●), 500 μm (▲), 750 μm (◆) and 1000 μm (■). (Inset) Concentration dependence of the pseudo‐first‐order rate constant of enzyme inactivation. (C) Time‐dependent inactivation of *Cb* GAPDH by GSSG, 2.5 mm (●), 5.0 mm (▲), 7.5 mm (◆) and 10 mm (■). (Inset) Concentration dependence of the pseudo‐first‐order rate constant of enzyme inactivation. Activities are given as a percentage of the initial activity (100 ± 5.3 U·mg^−1^) before the inactivation experiment. The results are presented as means of at least three independent experiments with standard deviations.

**Figure 3:**
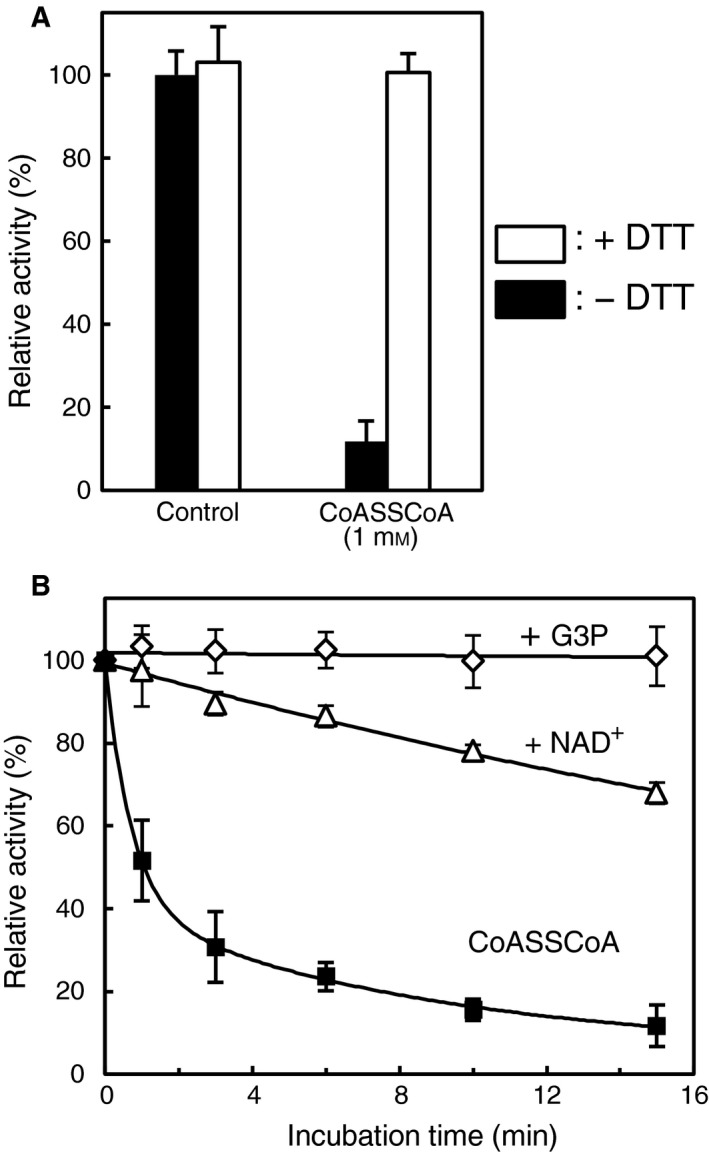
Reversibility of *Cb* GAPDH by DTT and effects of the substrate and cofactor on inactivation of *Cb* GAPDH. (A) Reversibility of the inactivated enzyme by DTT. The *Cb* GAPDH inactivated by 1 mm CoASSCoA for 15 min was incubated with 10 mm DTT for 15 min, and then, residual activity was assessed before (black bar) and after (white bar) treatment of DTT. The control experiment (without any treatment) is given for comparison. (B) The effects of G3P or NAD ^+^ during inactivation treatment. The enzyme was preincubated with 2 mm G3P (♢) or 1 mm NAD ^+^ (▵) for 5 min and then treated with 1 mm CoASSCoA for 0–15 min. Inactivation curve of 1 mm CoASSCoA is presented for comparison (■). Activities are given as a percentage of the initial activity (100 ± 5.3 U·mg^−1^) before the inactivation experiment. The results are presented as means of at least three independent experiments with standard deviations.

Identification of *Cb*GAPDH as a CoAlated target under NaOCl stress *in vivo* prompted us to investigate the redox regulation of *Cb*GAPDH activity by CoAlation *in vitro*. To examine whether *Cb*GAPDH undergoes CoAlation or not, we treated the enzyme with coenzyme A disulphide (CoASSCoA), because GSSG and CoASSCoA were used to test protein S‐thiolation 6, 23, 34. In this study, in order to clearly observe detectable changes in the enzyme activity, the effect of CoASSCoA was examined at the high concentration of 1 mm CoASSCoA, which is unlikely to occur in bacterial cells. Incubation of *Cb*GAPDH with 1 mm CoASSCoA caused enzyme inactivation in a time‐ and concentration‐dependent manner (half‐time of inactivation; *t*_1/2 _= 1.07 min) (Fig. 2A,B and Table 3). Incubation with buffer alone or 1 mm CoA as control caused no significant change in the activity within the incubation time. The CoASSCoA‐mediated inactivation could follow pseudo‐first‐order kinetics in the range of 250–1000 μm, with an apparent second‐order rate constant of 3.0 ± 0.28 m ^−1^·s^−1^. For GSSG‐dependent inactivation, we also obtained an apparent second‐order rate constant of 0.34 ± 0.029 m ^−1^·s^−1^, which is 10‐fold lower than that of CoASSCoA (Fig. 2C). About 10 mm GSSG was required for the complete loss of enzyme activity (*t*_1/2 _= 3.45 min). The inactivated enzyme activity caused by CoASSCoA treatment was almost fully recovered after treatment with 10 mm DTT, which suggested that reversibility of CoASSCoA‐dependent inactivation occurred via CoAlation (Fig. 3A). The substrate (G3P) or cofactor (NAD^+^) presence affected the inactivation profile (Fig. 3B and Table 3). The presence of G3P fully protected *Cb*GAPDH from inactivation, which suggested that the putative modification site is Cys149 because this cysteine forms a covalent thiohemiacetal intermediate with G3P during catalysis 24. NAD^+^ showed a slight protective effect against inactivation presumably because of the steric hindrance of NAD^+^ for the access of bulky CoASSCoA (*t*_1/2 _= 28.08 min). Similarly, S‐glutathionylation of *Cb*GAPDH by 10 mm GSSG was fully prevented by G3P (data not shown), and the inactivation was delayed in the presence of NAD^+^ (Table 3).

**Table 3:** Half‐time of inactivation treatment to *Cb*GAPDH. The data show the incubation times to give 50% activities after various inactivation treatments

Conditions	Without NAD^+^ (min)	With NAD^+^ (min)
1 mm CoASSCoA	1.07	28.1
0.1 mm H_2_O_2_	2.04	3.56
0.1 mm H_2_O_2_ + 1 mm CoA	2.02	3.62
10 mm GSSG	3.45	28.2
0.1 mm H_2_O_2_ + 1 mm GSH	2.22	2.70

**Figure 4:**
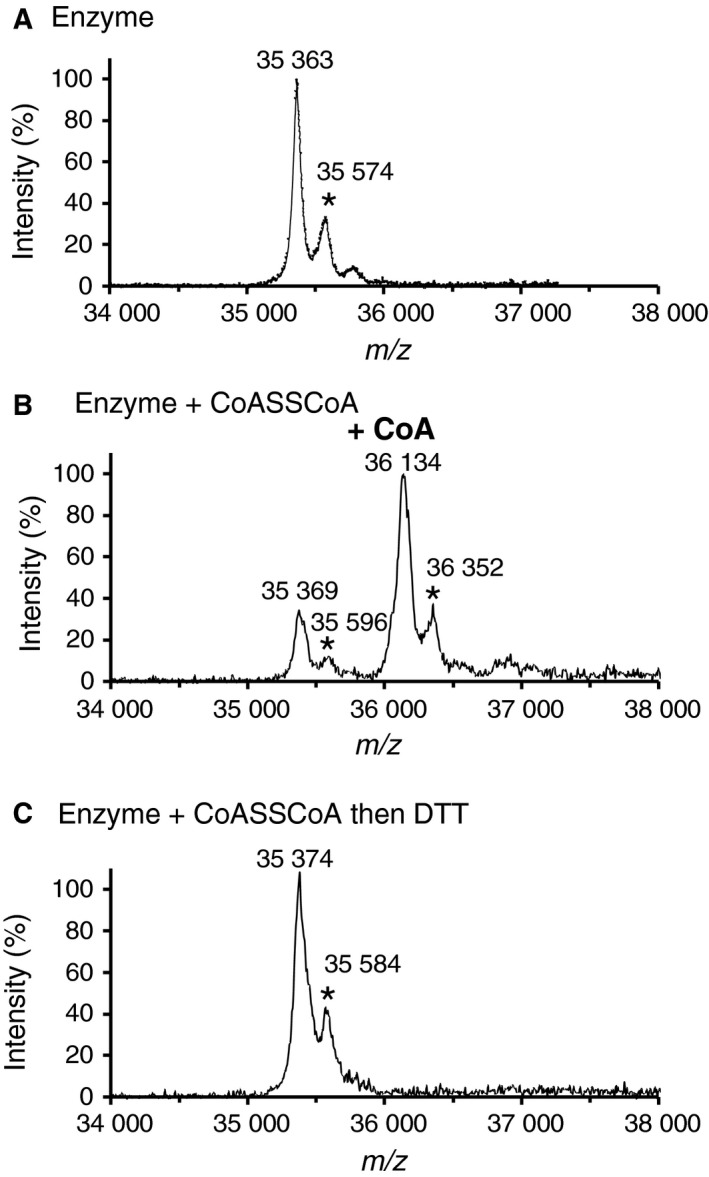
MALDI‐TOF mass spectra of *Cb* GAPDH treated with CoASSCoA. (A) The spectrum of native enzyme was acquired without any treatment. The spectra of *Cb* GAPDH incubated with 1 mm CoASSCoA (30 min) were acquired before (B) and after (C) the treatment with 10 mm DTT (30 min). The signals marked by * are attributed to the adducts of sinapinic acid.

CoAlation of *Cb*GAPDH was evidenced by MALDI‐TOF‐MS (Fig. 4). The apparent peak before the treatment with CoASSCoA (35 363 Da) was assigned to that of native *Cb*GAPDH. A mass increase of 771 Da, similar to the molecular weight of CoA (767.5 Da), was observed after the treatment with CoASSCoA (36 134 Da), and this was totally reversed by DTT (35 374 Da). These results indicate that *Cb*GAPDH was covalently modified by one CoA molecule per subunit, and the mixed disulphide between CoA and the enzyme was reduced by DTT. As shown by the relative intensity of the mass spectrum, approximately 70% of the subunits (3 subunits of tetramer) underwent CoAlation.

Altogether, the CoASSCoA treatment experiment showed that *Cb*GAPDH underwent CoAlation, and resulted in reversible enzyme inactivation that involved covalent attachment of CoA to Cys149 of the enzyme.

### Identification of CoAlated cysteine of *Cb*GAPDH by mass spectrometry

**Figure 5:**
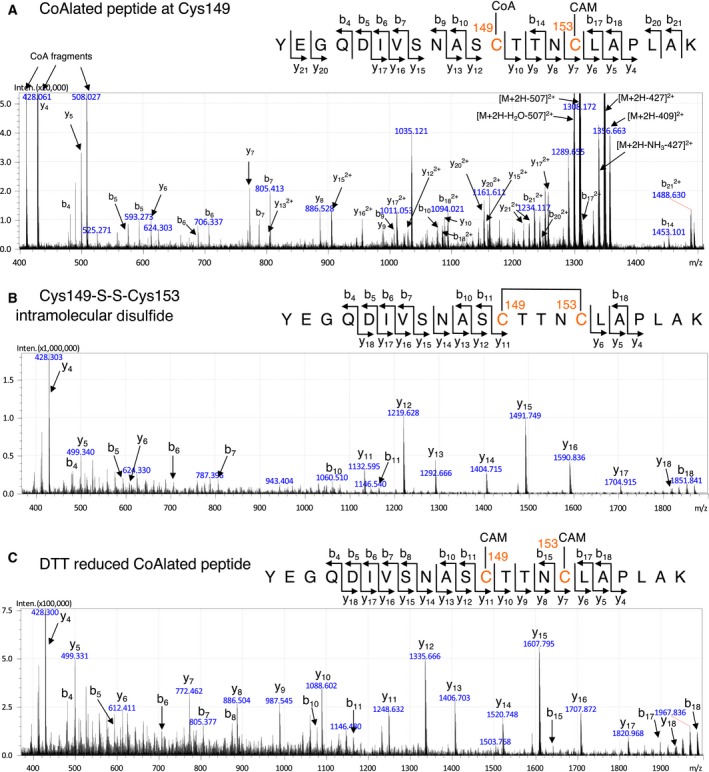
Identification of modification sites of *in vitro* CoAlated *Cb* GAPDH by using LC‐MS/MS. (A) The fragmentation pattern of MS/MS spectrum indicates that active‐site Cys149 is CoAlated, while Cys153 is carbamidomethylated. (B) Intramolecular disulphide bond between Cys149 and 153 was also detected as an additional redox modification of *Cb* GAPDH. (C) These redox modifications were reversed in a DTT sensitive manner, which suggested the reversibility of these redox post‐translational modifications.

The above *in vitro* experimental results suggested that the modification site of *Cb*GAPDH by CoAlation should be the active‐site Cys149, which was verified by peptide mass fingerprinting and LC‐MS/MS analysis of *in vitro* CoAlated *Cb*GAPDH (Fig. 5, Fig. S3). In the spectra of peptide mass fingerprinting, the clear mass shifts were observed before and after incubation of native *Cb*GAPDH with IAM, which suggested that all 3 cysteines in *Cb*GAPDH were exclusively carbamidomethylated under our experimental conditions and there could be no artificial thiol‐disulphide exchange reaction after IAM treatment to the enzyme (Fig. S3A,B). From the mass spectra of CoAlated *Cb*GAPDH, the peaks of intramolecular disulphide bond (Cys149‐S‐S‐Cys153) and CoA‐ and CAM‐modified peptide containing Cys149 and Cys153 were observed, while Cys288 remains largely carbamidomethylated (Fig. S3C). These peaks of redox modifications disappeared after incubation with DTT, which suggested the reversal of CoAlation and intramolecular disulphide to CAM‐modified peptides by DTT (Fig. S3D). Moreover, the precise modification site by CoAlation was confirmed by MS/MS analysis. Consistent with the results of peptide mass fingerprinting, both a CoA‐ and CAM‐modified peptide and an intramolecular disulphide peptide were detected from CoAlated *Cb*GAPDH, and the modification sites were clearly annotated (Fig. 5A,B, Fig. S4). On the basis of the characteristic fragmentation of y‐ion and b‐ion series, the MS/MS spectrum clearly showed that Cys149 was CoAlated but Cys153 and Cys288 were carbamidomethylated. These CoAlated peptide and intramolecular disulphide bonding were fully reversed to CAM‐modified peptide by DTT, which is comparable to the spectrum of CAM‐modified native *Cb*GAPDH (Fig. 5C, Fig. S5). Therefore, the spectra of the CoAlated *Cb*GAPDH with/without DTT treatment firmly established that DTT‐reversible modification by CoAlation occurred at Cys149 of the enzyme, while the other Cys153 and Cys288 were carbamidomethylated. The results of mass spectrometry clearly demonstrated that *Cb*GAPDH is redox‐regulated by reversible modification on the active‐site Cys149 by CoAlation.

### Inactivation of *Cb*GAPDH by H_2_O_2_ and/or CoA

We next investigated the sensitivity of *Cb*GAPDH to H_2_O_2_ and reactive oxygen species‐mediated CoAlation *in vitro*. Besides thiol‐disulphide exchange mediated by CoASSCoA, thiol oxidation to sulphenate (‐SO^−^) is also relevant to S‐thiolation of GAPDH. Typically, oxidation of the active‐site cysteine (Cys149) of GAPDH yields sulphenate and subsequently yields sulphinate ($-{\text{SO}}_{2}^{-}$ ) or sulfonate ($-{\text{SO}}_{3}^{-}$ ) by overoxidation 32, 33, 34. In contrast to the latter two forms, sulphenate is a transient intermediate towards the formation of intramolecular disulphide or mixed disulphides with cellular low‐molecular‐weight thiols such as GSH, which results in reversible inactivation to prevent irreversible oxidation of GAPDH 4, 27, 28, 32, 33, 34. We thus examined the effects of H_2_O_2_ with/without CoA on *Cb*GAPDH activity to investigate whether CoA can protect *Cb*GAPDH against irreversible oxidation by CoAlation.

**Figure 6:**
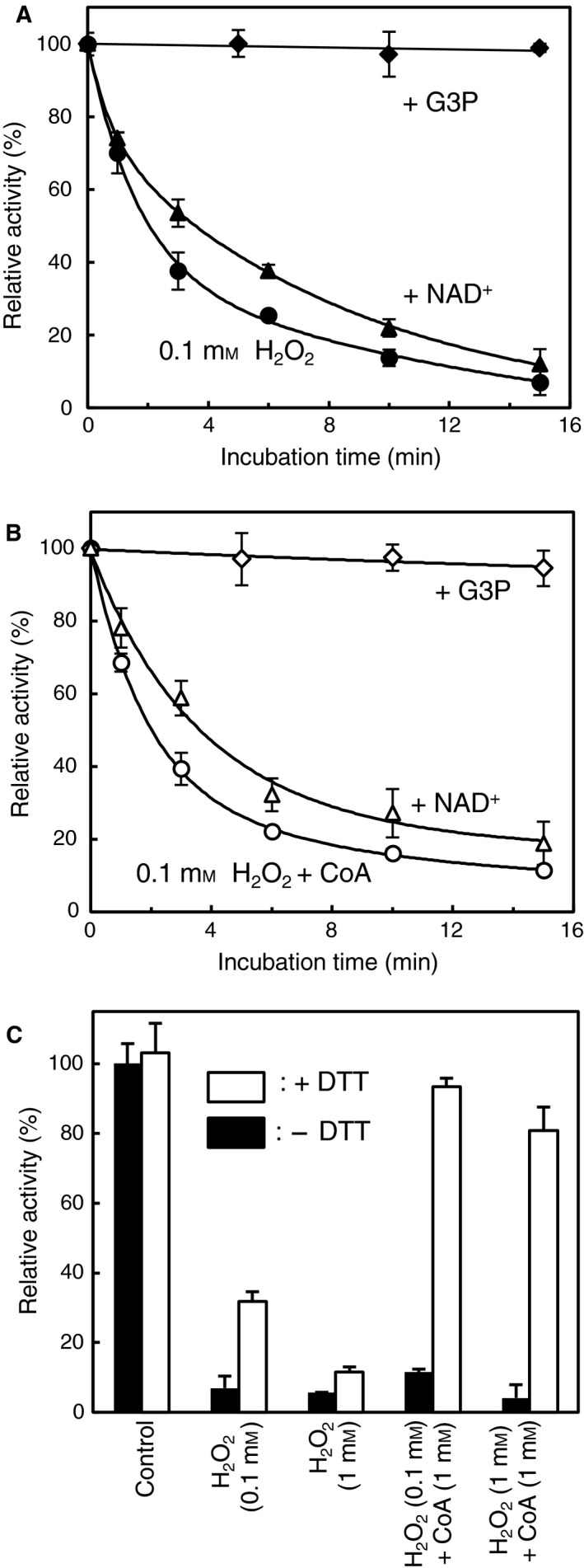
Inactivation profile of *Cb* GAPDH by H_2_O_2_ with/without CoA and the reversibility by DTT. (A) Time‐course inactivation of *Cb* GAPDH by H_2_O_2_ with/without G3P or NAD ^+^. The enzyme was incubated with only 0.1 mm H_2_O_2_ (●), plus 2 mm G3P (◆) or plus 1 mm NAD ^+^ (▲) for 0–15 min. G3P or NAD ^+^ was preincubated with enzyme for 5 min before the treatment with H_2_O_2_. (B) Time‐course inactivation of *Cb* GAPDH by H_2_O_2_ plus CoA with/without G3P or NAD ^+^. The enzyme was incubated with only 0.1 mm H_2_O_2_ and 1 mm CoA (○), plus 2 mm G3P (♢) or plus 1 mm NAD ^+^ (▵) for 0–15 min. CoA, G3P and/or NAD ^+^ were preincubated with the enzyme for 5 min before the treatment with H_2_O_2_. (C) Reversibility of the inactivated enzyme by DTT. The *Cb* GAPDH inactivated at indicated conditions for 15 min was incubated with 10 mm DTT for 15 min, and then, residual activity was assessed before (black bar) and after (white bar) treatment with DTT. The control experiment (without any treatment) is given for comparison. Activities are given as a percentage of the initial activity (100 ± 5.3 U·mg^−1^) before the inactivation experiment. The results are presented as means of at least three independent experiments with standard deviations.

**Figure 7:**
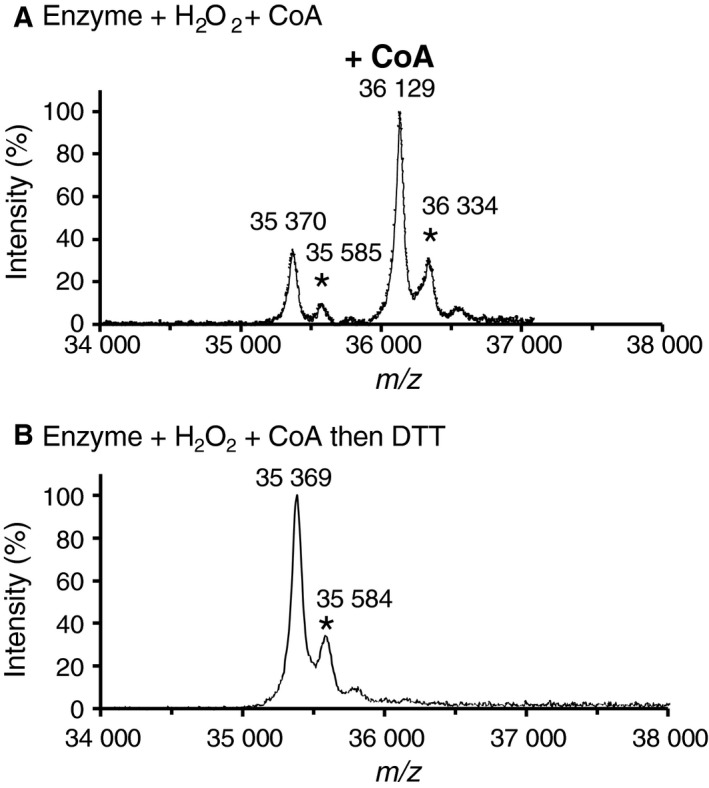
MALDI‐TOF mass spectra of *Cb* GAPDH treated with H_2_O_2_ plus CoA. The spectra of the enzyme incubated with 0.1 mm H_2_O_2_ plus 1 mm CoA (30 min) were acquired before (A) and after (B) the treatment with 10 mm DTT (30 min). The signals marked by * are assigned to the adducts of sinapinic acid.

The enzyme activity was almost completely lost after treatment with 0.1 mm H_2_O_2_ (*t*_1/2 _= 2.04 min), which resulted in irreversible inactivation (Fig. 6A). The activity of inactivated enzyme by 0.1 and 1 mm H_2_O_2_ could be recovered only to 32% and 12% of its original activity, respectively, by addition of 10 mm DTT (Fig. 6C). *Cb*GAPDH may mostly undergo irreversible oxidation ($-{\text{SO}}_{2}^{-}$ , $-{\text{SO}}_{3}^{-}$), whereas only part of *Cb*GAPDH undergoes reversible oxidation that can be recovered by DTT *in vitro*. In agreement with enzyme assay, we could detect the intramolecular disulphide bonding (Cys149‐S‐S‐Cys153) as by‐product after H_2_O_2_ treatment in addition to overoxidised peptide at Cys149 by using LC‐MS/MS (Fig. S6). The result of mass spectrometry well explains why the activity of H_2_O_2_‐treated *Cb*GAPDH is partially reactivated after DTT treatment. The treatment of 0.1 mm H_2_O_2_ in the presence of 1 mm CoA also caused enzyme inactivation (*t*_1/2 _= 2.02 min) with a comparable inactivation curve to 0.1 mm H_2_O_2_ (Fig. 6B). However, decreased enzyme activity by 0.1 and 1 mm H_2_O_2_ plus CoA could be recovered to 93% and 81% by DTT, respectively (Fig. 6C), which suggested that the enzyme undergoes reversible inactivation presumably via CoAlation. Indeed, similar to CoASSCoA‐dependent CoAlation, the observed mass shift (766 Da) after the treatment of H_2_O_2_ plus CoA (36 129 Da) was totally reversed by DTT (35 369 Da; Fig. 7), which indicated that *Cb*GAPDH was covalently modified by a CoA molecule per subunit in a reversible manner.

**Figure 8:**
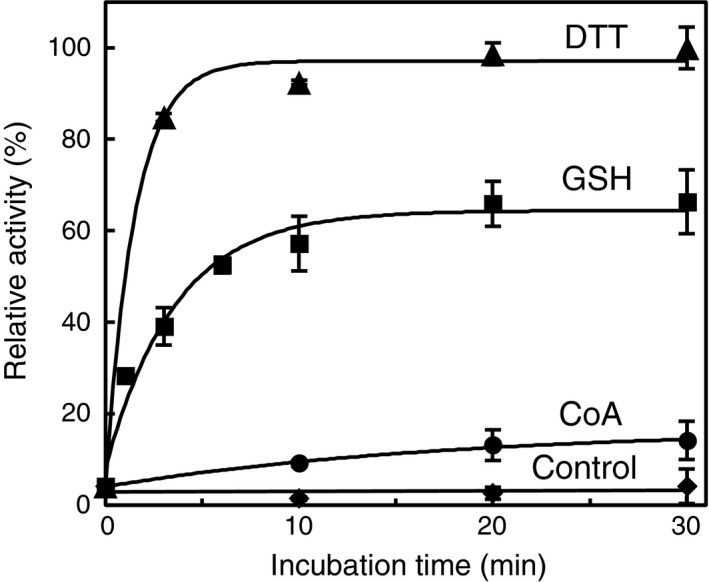
Time‐course reactivation of CoAlated *Cb* GAPDH by thiol compounds. The CoAlated enzyme was incubated with 10 mm DTT (▲), 5 mm GSH (■), 1 mm CoA (●) and buffer alone (◆) for 0–30 min, and the remaining activity was assessed. Activities are given as a percentage of the maximum activity measured after 30 min incubation with 10 mm DTT (93.5 ± 5.0 U·mg^−1^). The results are presented as means of at least three independent experiments with standard deviations.

We also examined whether GSH and CoA, cellular low‐molecular‐weight thiols, can reduce the CoAlated *Cb*GAPDH and reactivate its glycolytic activity *in vitro* (Fig. 8). Nonenzymatic dethiolation mediated by 5 mm GSH was effective (66%), whereas 1 mm CoA had little effect on dethiolation of the enzyme (14%). Preincubation with G3P before treatment of H_2_O_2_ with/without CoA to *Cb*GAPDH fully prevented enzyme inactivation because a covalent thiohemiacetal intermediate with G3P could prevent cysteine oxidation to sulphenate and its subsequent reaction with CoA by CoAlation (Fig. 6A,B). In contrast, the protective effect of NAD^+^ was slight (*t*_1/2 _= 3.56 and 3.62 min). The formation of labile sulphenate intermediate at Cys149 was confirmed by LC‐MS/MS with aid of dimedone trapping technique (Fig. S6), which is widely used to identify the formation of protein sulphenate 42. Therefore, the unstable sulphenate at Cys149 can be transient intermediate towards protein CoAlation. For comparison, inactivation of *Cb*GAPDH by S‐glutathionylation in the presence of 0.1 mm H_2_O_2_ plus 1 mm GSH with/without NAD^+^ also gave a similar inactivation curve (Table 3). These results suggest that *Cb*GAPDH readily undergoes irreversible inactivation by H_2_O_2_, which can be efficiently prevented by CoAlation in the presence of H_2_O_2_ and CoA *in vitro*.

### Inactivation of *Cb*GAPDH by NaOCl and/or CoA

Because *in vivo Cb*GAPDH CoAlation was observed under NaOCl stress, we also examined the sensitivity of *Cb*GAPDH activity after NaOCl exposure, and CoAlation of *Cb*GAPDH with NaOCl and CoA to confirm whether strong oxidising agent NaOCl can induce CoAlation of the enzyme *in vitro*. Hypochloric acid (HOCl) has high reactivity with Cys thiol with second‐order rate constant of 3 × 10^7^ m ^−1^·s^−1^ 43. Therefore, redox active thiols readily undergo overoxidation pathway via unstable sulphenylchloride (–SCl) intermediate, which is very rapidly overoxidised to sulphinate or sulfonate 27, 28, 43, whereas the irreversible overoxidation of chlorinated Cys can be prevented through S‐thiolation pathway by the formation of mixed disulphide with low‐molecular‐weight thiols. *Cb*GAPDH was quite sensitive to NaOCl‐mediated overoxidation, which led to irreversible enzyme inactivation *in vitro*, whereas the treatment with NaOCl in the presence of CoA caused reversible enzyme inactivation (Fig. S7). The reversal of the *Cb*GAPDH activity by DTT and MALDI‐TOF mass spectra indicates that the enzyme activity is redox‐regulated by DTT‐reversible binding of CoA to the enzyme by CoAlation under NaOCl stress *in vitro* (Figs S7 and S8). Similar to H_2_O_2_‐mediated *Cb*GAPDH inactivation, we could detect both intramolecular disulphide bonding (Cys149‐S‐S‐Cys153) and overoxidised peptide at Cys149 after NaOCl treatment by LC‐MS/MS (Fig. S9). Overall, *Cb*GAPDH is highly susceptible to overoxidation by H_2_O_2_ or NaOCl, which can be efficiently prevented by the formation of mixed disulphide between the enzyme and CoA *in vitro*. We thus confirmed that *Cb*GAPDH is redox‐regulated and protected against overoxidation by protein CoAlation at Cys149 under both H_2_O_2_ and NaOCl stress *in vitro*.

### Titration of free thiols in native, oxidised, and CoAlated *Cb*GAPDH

**Figure 9:**
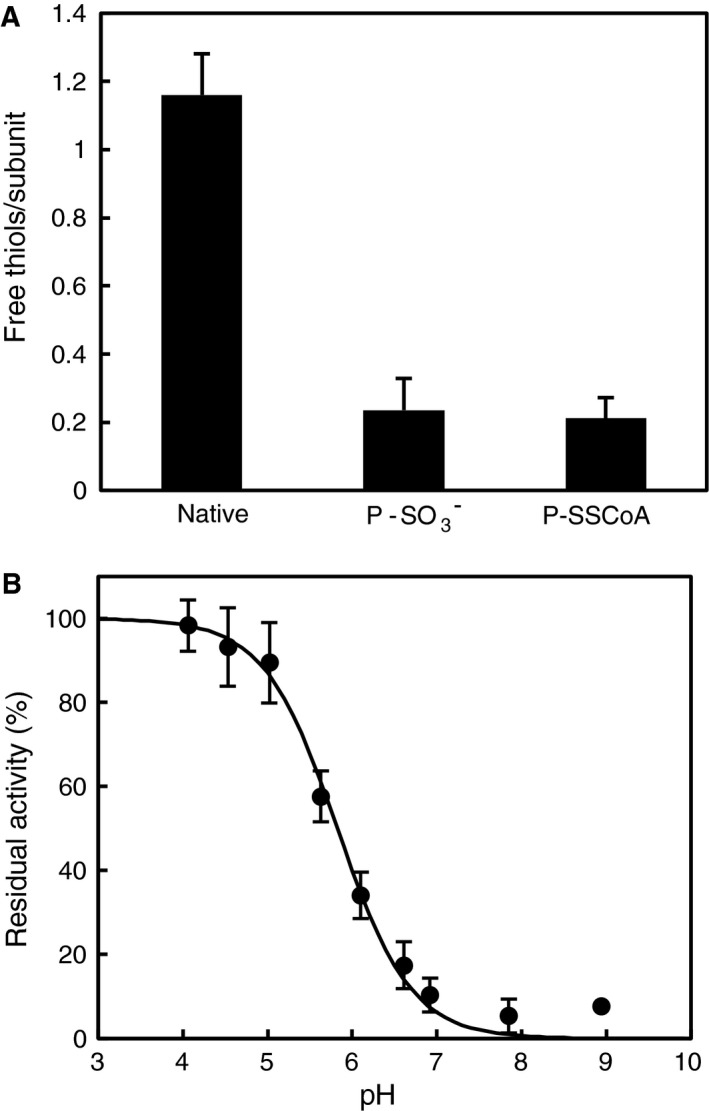
Quantification of free thiols and pKa determination. (A) The number of free thiols in native, oxidised and CoAlated *Cb* GAPDH. The absorbance at 412 nm of the solution after the reaction with free thiols in *Cb* GAPDH and DTNB was measured by UV–vis. (B) pKa titration curve of the active‐site Cys149 in *Cb* GAPDH. The pKa was estimated to be 5.82 ± 0.05 by fitting each point to a derivation of the Henderson–Hasselbalch equation. The results are presented as means of at least three independent experiments with standard deviations.

To clarify the number of cysteine residues modified by oxidation or CoAlation in *Cb*GAPDH, the number of free thiols in native, oxidised and CoAlated *Cb*GAPDH was quantified spectrophotometrically by using 5, 5′‐dithiobis(2‐nitrobenzoic acid) (DTNB) (Fig. 9A). The free thiols in native enzyme (1.16 ± 0.12) decreased after oxidation (0.24 ± 0.09) and CoAlation (0.21 ± 0.06) of the enzyme, which indicated that a cysteine per subunit was lost after oxidation or CoAlation. Among three cysteine residues (Cys149, Cys153 and Cys288) found in the amino acid sequence of *Cb*GAPDH (Fig. S2), the active‐site Cys149 should be the only accessible cysteine in *Cb*GAPDH because it is located in a p*K*_a_‐lowering environment near His176. Indeed, the p*K*_a_ of Cys149 determined by iodoacetamide (IAM) titration was 5.82 ± 0.05 (Fig. 9B), which is comparable with GAPDHs from *E*. *coli* (5.70) 44, *G*. *stearothermophilus* (5.92) 44 and *Arabidopsis* *thaliana* (5.65) 33. In contrast, the other cysteine residues (Cys153 and Cys288) may remain unreactive with DTNB because of steric constraints or not being a redox‐sensitive cysteine. Overall, the results indicated that the active‐site Cys149 of *Cb*GAPDH can be a putative modification site for protein CoAlation.

### Computational docking model of CoAlated *Cb*GAPDH

**Figure 10:**
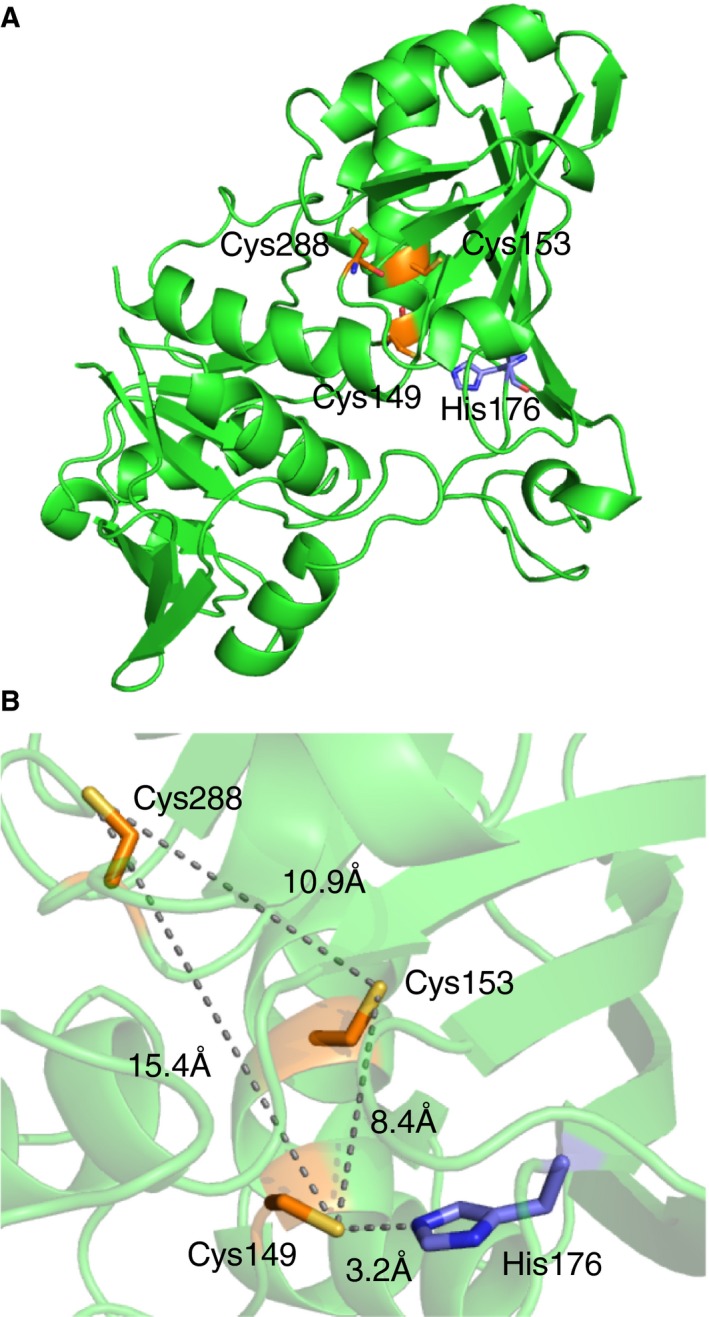
Homology‐modelled structure of *Cb* GAPDH. (A) Overall and (B) active‐site structure of apo *Cb* GAPDH. Cys149, Cys153, Cys288 and His176 are depicted by orange and violet sticks. The broken lines represent the distances between two sulfur or nitrogen atoms.

The results above showed that the target of CoAlation is likely to be the active‐site Cys149, which was also investigated by using a molecular docking approach. To clarify the potential conformational alternation of *Cb*GAPDH by CoAlation, far‐UV CD spectra of the enzyme before and after CoAlation were measured. The CD analysis revealed that CoAlation of *Cb*GAPDH did not accompany significant secondary structural change (Fig. S10). We also confirmed that *Cb*GAPDH retained its tetrameric structure after CoAlation as evidenced by gel filtration chromatography (data not shown). We thus used the homology‐modelled structures based on *E*. *coli* GAPDH as a template to predict the docking pose of CoAlated *Cb*GAPDH. The homology models of apo and holo (NAD^+^ bound) form of *Cb*GAPDH were built by using modeller (v9.15) program (University of California, San Francisco, CA, USA) (Fig. 10). The modelled structures of both apo and holo *Cb*GAPDH were expected to have 326 residues (99.4%) in favoured or allowed regions and two residues (0.6%) in outlier regions according to the accuracy evaluation by the RAMPAGE program, which indicated the reliability of the modelled structures. Homology modelling of *Cb*GAPDH showed that Cys149, nearby Cys153 at the same alpha‐helix structure and Cys288 had opposite directions and were far distant from each other (8.4–15.4 Å) to form intramolecular disulphide, which suggested that the formation of disulphide is unfavourable because it requires drastic rearrangement of the secondary structure near the active site. Indeed, conformational rigidity of GAPDH was also demonstrated in previous work 45.

**Figure 11:**
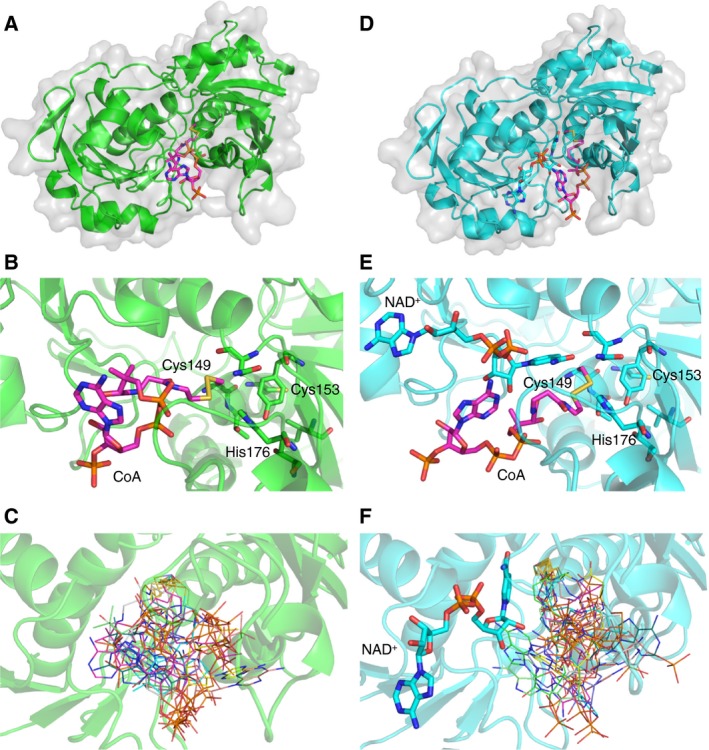
Molecular docking of *Cb* GAPDH and CoA by using the flexible side chain method in autodock4. The best poses of the modelled covalent complex between CoA and Cys149 of apo (A–C) or holo (D–F) *Cb* GAPDH are shown. The estimated free energies of the best binding pose were −10.37 and −8.38 kcal·mol^−1^ for the apo and holo structures, respectively. CoA is depicted in pink in the overall docking pose (A, D) and active site (B, E). The 10 best poses were overlaid and depicted in lines with different colours (C, F).

To model the pose of the disulphide bond between Cys149 and CoA, covalent docking was employed by using the flexible side chain method implemented in autodock4 46. The results clearly showed that both apo and holo enzymes could readily form mixed disulphides with CoA at Cys149 without steric constraints in the vicinity of the active site (Fig. 11A,B,D,E), although the conformational variations of CoA were reduced in the holo enzyme because of occupation of NAD^+^ (Fig. 11C,F and Fig. S11).

## Discussion

Protein CoAlation is emerging as an alternative redox post‐translational modification observed during oxidative or metabolic stress in mammalian cells and bacteria, or spore formation of *Bacillus cereus* with preference of metabolic enzymes 6, 18, 23. Among them, GAPDH was identified as a target of protein CoAlation in mammalian cell treated with H_2_O_2_ and *S. aureus* under diamide stress 6, 23. Protein CoAlation was highly induced when the cells are exposed to a strong oxidant NaOCl because of its fast reaction rate with Cys 43. In the present study, we revealed that *Cb*GAPDH is CoAlated under NaOCl stress *in vivo*. Besides protein CoAlation, intramolecular disulphide bonding could also contribute to the redox regulation of *Cb*GAPDH. In previous redox proteomic studies of *E. coli* and *Bacillus subtilis*, GAPDH was identified as target of intramolecular disulphide bond formation or S‐thiolation under H_2_O_2_ or NaOCl stress 30, 47 S‐thiolation has rational role for thiol protection against overoxidation to prevent the requirement of new protein synthesis of overoxidised protein for the recovery of the enzyme activity, and for regulating enzyme activity or functions by modifications of regulatory cysteines 1. The oxidative stress might cause growth delay and downregulation of glycolysis by inhibiting GAPDH activity by redox modification of conserved active‐site cysteine 30, 47. S‐thiolation of GAPDH and its subsequent inhibition of glycolytic activity can divert central carbon flux to oxidative pentose phosphate pathway (PPP) to supply high demand of NADPH as reducing power under oxidative stress as previously demonstrated 29, 39. Therefore, inhibition of GAPDH activity by S‐thiolation serves as a mechanism for this metabolic reconfiguration to mediate cellular antioxidant response.

**Figure 12:**
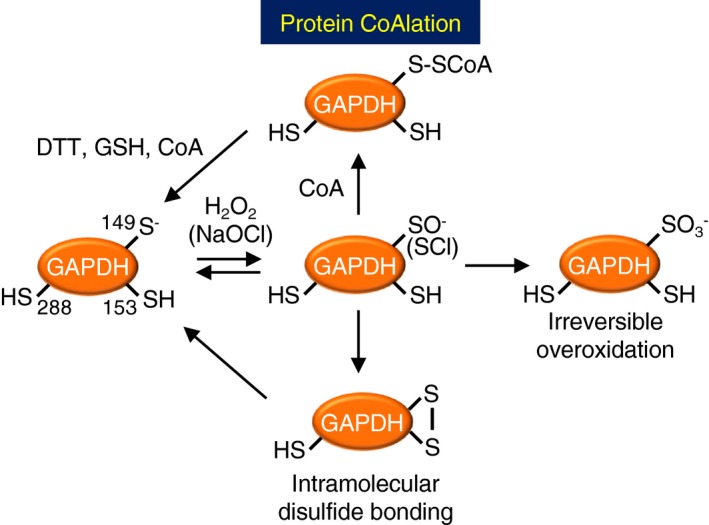
Schematic illustration of the CoAlation and de‐CoAlation mechanisms of *Cb* GAPDH. The active‐site Cys149 of *Cb* GAPDH forms cysteine sulphenate or sulphenyl chloride that further undergo overoxidation to sulfonate or intramolecular disulphide bonding in the presence of H_2_O_2_ or NaOCl alone, which resulted in irreversible inactivation of the glycolytic activity. While, *Cb* GAPDH is protected against this irreversible overoxidation by protein CoAlation and intramolecular disulphide bonding in the presence of H_2_O_2_/NaOCl and CoA. These modifications resulted in reversible enzyme inactivation due to CoAlation of Cys149.

We further demonstrated that the activity of *Cb*GAPDH is redox‐regulated and its active‐site Cys149 is protected against irreversible overoxidation by protein CoAlation under both H_2_O_2_ and NaOCl stress *in vitro*, which supports our *in vivo* findings (Fig. 12). Previous studies have examined the effects of CoASSCoA on the activity of enzymes *in vitro*. For instance, 3‐hydroxymethylglutaryl coenzyme A reductase was rapidly (*t*_1/2 _< 2 min) and completely inactivated reversibly with 1.5 μm CoASSCoA 48, and the activity of phosphofructokinase was reduced to 15% with > 2 h incubation of 46 μm CoASSCoA 49. Mammalian creatine kinase, GAPDH, isocitrate dehydrogenase and pyruvate dehydrogenase kinase were also reversibly inactivated by CoASSCoA, with varying extents of inactivation 6. In our case, the treatment with CoASSCoA even at micromolar concentrations led to inactivation of *Cb*GAPDH, whereas 10 mm GSSG, which is much higher than the physiological concentration, was required for complete inactivation of the enzyme. Consistently, it has been shown that the physiological concentration of GSSG is unlikely to lead to S‐glutathionylation of GAPDH 6, 33, 34. Although the significance of a CoASSCoA‐mediated mechanism *in vivo* remains unclear, accumulation of an intercellular concentration of CoASSCoA under oxidative stress would be a cause of CoAlation of *Cb*GAPDH. The determination of CoASSCoA concentration *in vivo* remains to be tackled.

Besides the CoASSCoA‐dependent mechanism, initial oxidation of labile cysteine to sulphenate followed by reaction with low‐molecular‐weight thiol is proposed as a plausible mechanism of S‐thiolation 25, 27, 32, 33, 34, 45. Indeed, *Cb*GAPDH undergoes CoAlation by incubation with H_2_O_2_ plus CoA. As revealed by MALDI‐TOF‐MS, approximately 70% of a single subunit of *Cb*GAPDH is modified by a CoA molecule after the treatment of CoASSCoA, or H_2_O_2_ plus CoA. This indicates that the major product after CoAlation is the mixed disulphide between *Cb*GAPDH and CoA. Regarding the examined protective effect by G3P, a thiol titration experiment and a covalent docking study, the active‐site Cys149 in *Cb*GAPDH is the putative modification site of CoAlation. Indeed, peptide mass fingerprinting and MS/MS analysis of *in vitro* CoAlated *Cb*GAPDH confirmed that active‐site Cys149 is CoAlated while Cys153 and Cys288 are carbamidomethylated. The intramolecular disulphide bonding (Cys149‐S‐S‐Cys153) was also detected as an alternative form of redox regulation of the enzyme. The formation of intramolecular disulphide bonding has been reported in several studies 28, 30, 32, 47. The structural determinant for the preference of these redox modifications (S‐thiolation or intramolecular disulphide) remains to be elucidated. Cys149 is known as a conserved cysteine for substrate binding and as the modification site of sulphenation and S‐thiolation of GAPDHs among all domains of life 27, 28, 33, 34, 45. In fact, the extreme vulnerability of Cys149 in *Cb*GAPDH to H_2_O_2_ was observed, which is consistent with the findings of previous studies 27, 28, 33, 34, 45. Overoxidised peptide at Cys149 could be detected by mass spectrometry after exposure of *Cb*GAPDH to H_2_O_2_ or NaOCl as main product *in vitro*. Dimedone trapped technique was employed to detect the target of sulphenation in *Cb*GAPDH treated with H_2_O_2_, further indicating transient formation of cysteine sulphenate and subsequent CoAlation at Cys149. This extremely high susceptibility of Cys149 to oxidation is explained by the existence of a conserved and dedicated specific H_2_O_2_ binding pocket, stabilisation of the reaction transient state and a proton relay mechanism promoting leaving group departure as demonstrated in human GAPDH 45, 50. Thus, the transient sulphenate upon oxidation should facilitate the subsequent reaction with thiols and result in a propensity towards S‐thiolation of GAPDH 45. The involved amino acid residues (Thr153, Cys156, Thr177 and Tyr314 in human GAPDH) for the proton relay mechanism and H_2_O_2_ sensitivity are also conserved in *Cb*GAPDH (Fig. S1). Owing to this mechanism, Cys149 of *Cb*GAPDH should be the major modification target of CoAlation, thereby preferentially preventing irreversible inactivation of the enzyme. GAPDH consists of two domains, namely, an N‐terminal Rossman‐fold (NAD binding) domain and a C‐terminal catalytic (substrate binding) domain 51. The Rossman‐fold binding motif is known to play a role in the interaction with NAD, while this motif was proposed to facilitate CoA binding to GAPDH as demonstrated in *Sa*GAPDH 23. In this model, the interaction between ADP moiety of CoA and vacant Rossman‐fold domain after NAD dissociation from subunits by overoxidation of catalytic cysteine may offer a transient binding mode towards the formation of mixed disulphide between CoA and active‐site cysteine of the enzyme.

In mammalian cells and tissue under oxidative or metabolic stresses, a number of key metabolic enzymes have been identified as CoAlated proteins in which GAPDH was one of the targets 6. The target cysteine residues of CoAlation in mammalian GAPDH were identified as Cys23 and Cys245, both of which are not conserved but are cysteine residues specific to mammalian GAPDH (Fig. S1) 6. It was demonstrated that *Sa*GAPDH is CoAlated at active‐site Cys151 and GapA2 isoform is CoAlated at noncatalytic Cys202 23. Therefore, the modification sites of CoAlation differ depending on the source of the enzyme, and it is likely that modifications at a different site would affect the enzyme activity and functions via discrete mechanisms. It has been demonstrated that dethiolation of GAPDH homologs can be driven nonenzymatically by cellular low‐molecular‐weight thiols or enzymatically by glutaredoxin (Grx), thioredoxin (Trx) or its analogous systems 27, 28, 32, 33, 36, 37, 38. For example, S‐glutathionylated chloroplastic A_4_‐GAPDH of *A. thaliana* was reactivated by Grxs but less efficiently so by Trxs or GSH 37. Cytosolic GAPDH of *A. thaliana* was deglutathionylated by both Grxs and Trxs 33. GSH or Trx catalysed deglutathionylation reactions of rabbit muscle GAPDH 32. Yeast GAPDH is dethiolated by Trxs but not by Grxs 38. There seems to be a wide diversity in redox‐controlled mechanisms of GAPDH homologs by S‐thiolation depending on the organisms, isoforms and conditions. In our case, GSH efficiently reduced CoAlated *Cb*GAPDH and regenerated the glycolytic activity *in vitro*, whereas CoA showed only a slight effect. The p*K*_a_ values of CoA (9.83) and GSH (8.93) indicated that the level of the reactive thiolate state is much higher in GSH compared with CoA at physiological pH 5. GSH is thus much more available as nucleophilic thiolate anion that can react with disulphides between protein thiols and cellular low‐molecular‐weight thiols. Consistent with this notion, the reduction of CoAlated *Cb*GAPDH is much more efficient in the presence of GSH, when compared to CoA *in vitro*. Under the reduced redox state, most thiol proteins exist as reduced thiol state in the cytoplasm as a result of the reductive potential exerted by the Trx or Grx systems 52. CoAlated protein that accumulated in the cell under oxidative stress could be reduced back to thiolate form after the recovery of the cells from oxidative stress. Thus, the intercellular redox state might be involved in the regulation of CoAlation of *Cb*GAPDH under oxidative stress. Whether or not dethiolation of CoAlated *Cb*GAPDH might be regulated by an enzymatic mechanism should be investigated in future studies.

In summary, we showed that CoAlation of *Cb*GAPDH functioned by protecting the active‐site Cys149 against irreversible overoxidation by H_2_O_2_ and NaOCl, thereby redox‐regulating enzyme activity. This is an alternative redox post‐translational modification for thiol protection and redox regulation of *Cb*GAPDH under oxidative stress in a manner analogous to S‐glutathionylation. More detailed studies will be required to understand the potential significance of CoAlation of GAPDH in redox signalling and metabolic regulation under oxidative stress.

## Materials and methods

### Reagents

All reagents were purchased from Sigma‐Aldrich (St. Louis, MO, USA) or Wako Pure Chem. Ind. Ltd. (Osaka, Japan) unless otherwise stated.

### Bacterial strain and culture condition

*Citrobacter* sp. S‐77 was grown in the modified Bacto marine medium as previously described 53. The cells that entered the stationary phase were harvested by centrifugation at 9000 ***g*** for 20 min. The harvested cells were washed once with 20 mm Tris/HCl (pH 7.5) and stored at −80 °C until use. For NaOCl treatment experiments, strain S‐77 was grown 37 °C in M9 minimal medium up to late‐logarithmic phase with an optical density at 500 nm (OD_500_) of 1.0 and then exposed to 500 μm NaOCl for 30 min. Bacterial growth was monitored by change of OD_500_ recorded by UV–vis spectrophotometer.

### Enzyme purification

The frozen cells (typically 15 g of wet weight) were thawed, suspended in 20 mm Tris/HCl (pH 7.5) with 1 mm DTT (buffer A) and disrupted by sonication at 60 W three times for 2 min in an ice bath by using an Ultrasonic Disruptor UD‐200 (TOMY SEIKO Inc., Tokyo, Japan). The cell debris and unbroken cell were removed by ultracentrifugation at 140 000 ***g*** for 60 min that used an Optima L‐90K Ultracentrifuge (Beckman Coulter Inc., Brea, CA, USA). All steps of enzyme purification were performed at 4 °C on  an AKTA‐FPLC system (GE Healthcare UK Ltd., Buckinghamshire, UK). The supernatant after ultracentrifugation was loaded onto a hydroxyapatite column (2.6 cm × 10 cm; Bio‐Rad Laboratories Inc., Hercules, CA, USA) pre‐equilibrated with buffer A at a flow rate of 7.0 mL·min^−1^. The column was washed with 2 column volumes of buffer A, and the enzyme was eluted by using a linear gradient of 0–0.6 m potassium phosphate (KP) (pH 7.5) containing 1 mm DTT over 6 column volumes. Active fractions were pooled, concentrated by using an Amicon Ultra‐15 (10000 NMWL; Millipore Corp., Burlington, MA, USA) and applied onto a Superdex 200 column (2.6 × 60 cm; GE Healthcare UK Ltd.) pre‐equilibrated with buffer A containing 150 mm NaCl at a flow rate of 3.0 mL·min^−1^. The fractions eluted as a single peak were pooled and stored at −80 °C until use. The protein purity was established by SDS/PAGE analysis using 12.5% acrylamide gels with standard molecular weight markers (GE Healthcare UK Ltd.) after staining in a dye solution of Coomassie Brilliant Blue R‐250. The molecular weight of the purified enzyme was estimated by gel filtration using a Superose 12 HR (10/300; GE Healthcare) column pre‐equilibrated with buffer A containing 150 mm NaCl. Standard molecular weight markers (Bio‐Rad Laboratories Inc.) containing bovine thyroglobulin (*M*_r_ = 670 000), bovine g‐globulin (*M*_r_ = 158 000), chicken ovalbumin (*M*_r_ = 44 000), horse myoglobin (*M*_r_ = 17 000), and vitamin B12 (*M*_r_ = 1350) were used for calibration. The N‐terminal amino acid sequence of the purified enzyme was determined by using an automated Edman degradation system consisting of an ABI protein sequencer 473A (Applied Biosystems, Tokyo, Japan). The protein band of the purified enzyme was made by using SDS/PAGE and then blotted onto a polyvinylidene difluoride membrane 54. Protein concentrations were measured routinely by using the established procedures of the Bio‐Rad Protein Assay (Bio‐Rad Laboratories Inc.) 55.

### Amino acid sequence alignment

The amino acid sequence of *Cb*GAPDH was compared with the deduced amino acid sequences of other GAPDHs available from the NCBI and GenBank databases. The multiple amino acid sequence alignment was performed by using the clustal w 2.1 program using the default parameters 56.

### Enzyme assay

The enzyme assays were performed in 100 mm Tris/HCl (pH 8.0) with 1 mm DTT, 40 mm KH_2_PO_4_, 1 mm NAD^+^ and 2 mm G3P at 30 °C. The formation of NADH was monitored at 340 nm (ε_340_ = 6220 m ^−1^·cm^−1^) using a JASCO V‐670 spectrophotometer. Typically, 60 s was used to determine the initial slope for the calculation of enzyme activity. One unit of activity was defined as the amount of enzyme catalysing the reduction of 1 μmol NAD^+^ per minute.

### Steady‐state kinetics

Steady‐state kinetic parameters were determined by fitting the data to the Michaelis–Menten equation with different concentrations of substrates (0–10, 0–1.0 and 0–40 mm for G3P, NAD^+^ and K_2_HPO_4_, respectively) in 100 mm Tris/HCl (pH 8.0) containing 1 mm DTT at 30 °C. The data were analysed by using an Enzyme Kinetics Module 1.1 of sigmaplot 8.0 software (Jandel Scientific, San Rafael, CA, USA) and nonlinear regression.

### Enzyme inactivation treatment

Before each experiment, the enzyme was desalted against 20 mm Tris/HCl (pH 7.5) with 150 mm NaCl by passing through a Sephadex G‐25 Hi Trap desalting column (GE Healthcare UK Ltd.) to remove free DTT in the solution. The buffer‐exchanged sample (typically 2.8 μm at final concentration) was incubated with H_2_O_2_ and/or CoA in 100 mm Tris/HCl (pH 7.5) at 25 °C. In time‐course experiments, aliquots were withdrawn periodically (0–15 min) at each indicated time to assess the residual enzyme activity. For subsequent recovery experiments, the inactivated enzyme was incubated with 10 mm DTT for 15 min at 25 °C. For kinetic analysis of CoASSCoA (0–1000 μm)‐ or GSSG (0–10 mm)‐mediated inactivation, the enzyme solution was incubated with various concentrations of reactants in 100 mm Tris/HCl (pH 7.0) at 25 °C, and remaining activity was assessed periodically for 0–20 min. The activity was assessed according to the method described in the enzyme assay, except that 1 mm DTT was omitted from the assay solution.

### Enzyme reactivation treatment

CoAlated or oxidised *Cb*GAPDH was prepared by incubating the enzyme with 0.1 mm H_2_O_2_ with/without 1 mm CoA for 30 min at 25 °C and subsequently desalted to remove remaining H_2_O_2_ and CoA. CoAlated *Cb*GAPDH (typically 2.8 μm at final concentration) was then incubated with thiol compounds in 100 mm Tris/HCl (pH 7.5) for 0–30 min at 25 °C, followed by assessment of the reactivated enzyme activity as described above.

### Quantification of free thiols

Oxidised or CoAlated *Cb*GAPDH was prepared as mentioned above. The free thiol content in native, oxidised and CoAlated *Cb*GAPDH was quantified spectrophotometrically by using DTNB (Ellman's reagent). The samples (6 μm) were incubated with 200 μm DTNB in 50 mm Tris/HCl (pH 7.5) for 20 min at 25 °C, followed by measurement of absorbance of DTNB at 412 nm (ε_412 _= 14 150 m ^−1^·cm^−1^) 57.

### Determination of p*K*_a_

The p*K*_a_ determination of *Cb*GAPDH was performed by cysteine titration with IAM as described previously 58. The enzyme (2.8 μm) was incubated with/without 300 μm IAM for 20 min at 25 °C in different buffers ranging from pH 4 to 9, followed by measurement of residual enzyme activity. The following buffers were used: 10 mm sodium acetate (pH 4–5), 10 mm MES (pH 5.5–6.5) and 10 mm Tris/HCl (pH 7–9). Then, the residual activity expressed as a percentage of maximal activity without IAM treatment was plotted against pH, and the p*K*_a_ value was calculated by fitting the data to a derivation of the Henderson–Hasselbalch equation 58.

### Sample preparation for mass spectrometry

For *in vivo* sample preparation, the NaOCl‐treated bacterium was collected and suspended in 100 mm Tris/HCl (pH 7.5) containing 150 mm NaCl and 100 mm iodoacetamide (IAM) for alkylating free thiols in proteins. The resulting suspension was sonicated, ultracentrifuged and subjected to purification of *Cb*GAPDH as described above, except that DTT was omitted throughout the procedure. Samples (*in vitro* CoAlated, overoxidised *Cb*GAPDH) were prepared by incubating DTT‐free *Cb*GAPDH with 1 mm CoASSCoA, 1 mm H_2_O_2_ or NaOCl in 50 mm Tris/HCl (pH 7.5) for 30 min. For sulphenic acid detection, the enzyme was incubated with 1 mm H_2_O_2_ in the presence of 10 mm dimedone for 30 min. For the reduction of the enzyme, 10 mm DTT was treated for 30 min before alkylation treatment. For *in vitro* sample preparation for mass spectrometry, *in vitro* prepared native, overoxidised, dimedone trapped and CoAlated *Cb*GAPDH were carbamidomethylated (CAM) by 10 mm IAM for 1 h at 25 °C in dark. The resulting *in vivo* and *in vitro* samples were separated by SDS/PAGE on 5–20% gradient gel under nonreducing condition, followed by staining with CBB. The bands of *Cb*GAPDH were excised and subjected to overnight in‐gel digestion with lysyl‐endopeptidase (Lys‐C) in 50 mm Tris/HCl (pH 8.5) at 37 °C. The digested peptides were extracted, and the resulting samples were subjected to peptide mass fingerprinting or LC‐MS/MS analysis after desalting and concentration of the sample using ZipTip C18 pipet tips (Millipore) following the manufacturer's instruction.

### MALDI‐TOF‐MS

The sample for measurement was desalted and concentrated by using ZipTip C4 (for whole protein) or C18 (for peptide) pipet tips (Millipore) and a solution of 50% acetonitrile and 0.1% trifluoroacetic acid following the manufacturer's instructions. Then, the sample was eluted by using a saturated matrix (sinapinic acid) solution with 50% acetonitrile and 0.1% trifluoroacetic acid and dropped onto a stainless steel target plate, and dried in air at room temperature. The mass spectra were acquired by AutoflexIII (Bruker Daltonics, Bremen, Germany) under the linear positive and negative mode. External mass calibration was performed by using (M + H) or (M − H) ions of the protein standard II or the peptide calibration standard II (Bruker Daltonics). Spectra of peptide mass fingerprinting were processed by using the multifunctional mass spectrometry software mmass 59.

### LC‐MS/MS analysis

Peptide samples were analysed by LCMS‐IT‐TOF system (Shimadzu, Kyoto, Japan). Liquid chromatography (LC) system consisted of a degasser (DGU‐20A5), binary pump (LC‐20AD), autosampler (SIL‐20AC), column oven (CTO‐20A) and UV detector (SPD‐20A). Chromatographic separation was performed by using HPLC packed column Shim‐pack VP‐ODS (150 × 2.0 mm) (Shimadzu). The mobile‐phase A was 0.05% (v/v) formic acid, and mobile‐phase B was acetonitrile with 0.05% (v/v) formic acid. The injected samples were eluted with a linear gradient of 0–80% B (0–30 min), 80% B (30–35 min) for washing column and 100% A (35–40 min) for equilibrium at a flow rate of 0.2 mL·min^−1^. The temperature of column oven was set at 40 °C. The eluent was monitored at 220 and 280 nm with UV detector. MS analysis was performed on ion trap time‐of‐flight mass spectrometry (IT‐TOF‐MS) equipped with an electrospray ionisation (ESI) source operated in positive mode. The operating conditions were as follows: interface voltage, 4.5 kV; detector voltage, 1.9 kV; heat block temperature, 200 °C; curved desolvation line temperature, 200 °C; nebulising gas flow, 1.5 L·min^−1^; drying gas (N_2_) pressure, 100 kPa. Spectra were acquired in positive mode by using data‐dependent acquisition (DDA) mode, which permits a switch automatically from MS1 to MS2 mode. In brief, the top three ions from each MS1 spectrum were selected as precursor ions for fragmentation in MS2 mode. MS1 spectrum acquisition was performed as follows: ion accumulation time, 25 ms; repeat, three times, scan range *m/z* 1000–1600. MS2 spectrum acquisition was performed under the conditions as follows: collision‐induced dissociation (CID) energy, 50%; collision gas, 50%; q value, 0.251(45 kHz); ion accumulation time, 50 ms; repeat, 20 times; mass range, *m/z* 50–2000. These parameters were optimised to obtain reliable MS2 spectra if necessary. If the spectrum was not reliable for identification of modification site by DDA mode, targeted mode was employed by focusing on MS2 of specific precursor ions from MS1 acquisitions. The resulting data were processed by using the multifunctional mass spectrometry software mmass 59, and then, the annotated y‐ion and b‐ion series of peptides were manually inspected. Valuable modifications were set to CAM (C, +57), propionamide (PA; C, +71), disulphide bond (C, −2), oxidation (C, +48), dimedone (C, +138), CoAlation (C, +765). The specific fragmentation of CoA (C, +258, 338, 356) was taken into consideration for the identification of y and b‐ion fragments of CoAlated peptides. The parameters for database searches were as follows: enzyme, Lys‐C; mass value, monoisotopic mass; mass tolerance, 0.5 Da; maximum missed cleavages, 1.

### Circular dichroism

CoAlated *Cb*GAPDH was prepared as mentioned above. Before the acquisition of spectra, the native or CoAlated enzyme solution was exchanged with a buffer of 10 mm KP (pH 7.5). The CD spectra of native and CoAlated enzyme (0.1 mg·mL^−1^) were recorded in the far‐UV (190–260 nm) region by using a Chirascan V‐100 CD spectrophotometer (Applied Photophysics Ltd., Surrey, UK) and a 0.1 cm path length cuvette at 25 °C. The data pitch was set to 0.2 nm in continuous scan at a speed of 50 nm·min^−1^ with a bandwidth of 1.0 nm. The CD spectrum of the buffer alone was recorded and subtracted from the sample spectrum. Each spectral line was plotted by the average of three scans.

### Protein homology modelling and molecular docking

Homology‐modelled structures of *Cb*GAPDH were constructed by using a protein homology modelling program modeller (v9.15) 60 based on the crystal structure of *E*. *coli* GAPDH (96% identity) obtained from the Protein Data Bank (PDB): apo form (PDB: 1DC5 chain A) and holo form containing NAD^+^ (PDB: 1DC6 chain A) 61. All water molecules and ions present in the crystallographic structures were removed before running the program. The accuracies of the resulting homology models were evaluated by Ramachandran plot analysis and the RAMPAGE program 62. The modelled structures were used for further molecular docking experiment by autodock (v4.2) 63. To model a pose of disulphide bond formation between the *Cb*GAPDH active‐site Cys149 and CoA, covalent docking was employed by using the flexible side chain method 46. autodocktools (v1.5.6) was used to process the ligand and receptors and to prepare the files for covalent docking experiment by following their procedure 63. For the ligand preparation, the 3D conformer structure of CoA was obtained from PDB. The ligand coordinate tethered with two receptor atoms was prepared, and its geometry was optimised by using avogadro (v1.2) 64. The atoms of the ligand were then superimposed on the corresponding atoms in the receptor to allocate covalent bond with the target residue of Cys149 in the protein active site. The resulting side chain–ligand complex was treated as flexible in the receptor, and its position and conformation were further optimised by using a standard flexible docking procedure implemented in autodock4. All hydrogen atoms were added to the rigid protein and flexible ligand, and atomic Gasteiger charges were calculated. The dimension of the grid was set to 100 × 100 × 100 points with a grid spacing of 0.25 Å, and the grid centre was set up to cover the active‐site cysteine and ligand and to allow the fully extended ligand to freely rotate within the grid box. Docking simulations were performed by using 100 runs for both apo and holo structures and the Lamarckian genetic algorithm, with all other parameters kept at their default values. The docking results were visualised by using the pymol molecular graphics system (version 1.7; Schrödinger, LLC, New York, NY, USA).

### Statistical analysis

All results are presented as means of at least three independent experiments with standard deviations.

## Author contributions

KT, K‐SY and SO conceived and designed the project; KT and K‐SY acquired, analysed and interpreted the data; and KT and K‐SY wrote the paper. All authors have read and approved the manuscript.

## Conflict of interest

The authors declare no conflict of interest.

## Supporting information


**Fig. S1.** SDS‐PAGE (12.5%) analysis of purified *Cb*GAPDH. Lane 1, low‐molecular‐weight standard marker proteins (MW, 14 400–97 000); lane 2, purified *Cb*GAPDH.

**Fig. S2.** Multiple sequence alignment of GAPDH homologs. The sequences of other GAPDHs were extracted from the protein database of NCBI (www.ncbi.nlm.nih.gov); *Citrobacter* sp. S‐77 GapA (GAN52675.1), *Citrobacter* sp. S‐77 GapC (WP_045442819), *Escherichia coli* (P0A9B2), *Staphylococcus aureus* (Q6GB58)*, Geobacillus stearothermophilus* (P00362)*, Streptococcus pyrogenes* (P0C0G6)*, Salmonella *Typhimurium (P0A1P0), *Arabidopsis thaliana* (P25856), *Homo sapiens* (P04406), *Oryctolagus cuniculus* (P46406) and *Rattus norvegicus* (P04797). The N‐terminal sequence of *Cb*GAPDH determined in this study is indicated by a red character. The residues conserved in all sequences are shown with a grey background, and the cysteine or conserved histidine residues are shown with yellow or white characters on a black background.

**Fig. S3.** Peptide mass fingerprinting of native and CoAlated *Cb*GAPDH *in vitro*. The mass spectra of native *Cb*GAPDH (A) and CAM treated *Cb*GAPDH (B) showed that the mass shift of 2CAM (114 Da) in Cys149 and 153 containing peptide, and 1CAM (57 Da) in Cys288 containing peptide. These results indicated that in our experimental conditions, incubation of the enzyme with 10 mm IAM for 60 min in dark was enough to totally proceed carbamidomethylation for alkylation of free cysteines in *Cb*GAPDH. (C) *In vitro* CoAlation of *Cb*GAPDH occurred in Cys149 and 153 containing peptide, while Cys288 was exclusively carbamidomethylated. Intramolecular disulphide bonding (Cys149‐S‐S‐Cys153) was also detected as an alternative redox regulatory mechanism. (D) These redox modifications were reversed in a DTT dependent manner since CAM modified Cys149 and 153 containing peptide was detected. Note that the spectrum of (C) was recorded with linear negative mode, while the other spectra were recorded in linear positive mode.

**Fig. S4.** MS/MS spectrum of *in vitro* CoAlated *Cb*GAPDH. Peptide with Cys288 remains carbamidomethylated after our CoAlation assay condition.

**Fig. S5.** MS/MS spectra of native *Cb*GAPDH. (A) Carbamidomethylated peptide at Cys149 and 153. (B) Carbamidomethylated peptide at Cys288.

**Fig. S6.** MS/MS spectra of *in vitro* overoxidised *Cb*GAPDH by 1 mm H_2_O_2_. (A) Dimedone trapped indicated the formation of labile intermediate sulphenate at Cys149, while Cys153 is carbamidomethylated. (B) Over‐oxidation of Cys149 was confirmed, while Cys153 is carbamidomethylated. (C) Cys149‐S‐S‐Cys153 intramolecular disulphide bonding was also detected.

**Fig. S7.** Inactivation of *Cb*GAPDH by NaOCl with/without CoA and the reversibility by DTT *in vitro*. *Cb*GAPDH was incubated at each indicated conditions in the buffer solution of 100 mm Tris/HCl (pH 7.5) at 25 °C for 15 min and subsequently treated with 10 mm DTT for 15 min. The residual activity was then determined before (black bar) and after (white bar) the treatment of DTT. The control experiment (without any treatment) is given for comparison. Activities are given as a percentage of the initial activity (100 ± 5.3 U·mg^−1^) before the inactivation experiment. The results are presented as means of at least three independent experiments with standard deviation.

**Fig. S8.** MALDI‐TOF mass spectra of *Cb*GAPDH treated with NaOCl plus CoA *in vitro*. The mass spectra of *Cb*GAPDH incubated with 0.1 mm NaOCl in the presence of 1 mm CoA (30 min) were acquired before (A) and after (B) the treatment with 10 mm DTT (30 min). The peaks marked by * is assigned to the adducts of sinapinic acid.

**Fig. S9.** MS/MS spectra of overoxidised *Cb*GAPDH by 1 mm NaOCl *in vitro*. (A) Over‐oxidation of Cys149 was confirmed, while Cys153 is carbamidomethylated. (B) Cys149‐S‐S‐Cys153 intramolecular disulphide bonding was also detected.

**Fig. S10.** Far‐UV CD spectra of native (black line) and CoAlated (red dotted line) *Cb*GAPDH. The spectra were recorded in 190–260 nm, and the data are presented as molar ellipticity. Each spectra line was plotted by the average of three scans.

**Fig. S11.** Modelled structures of CoAlated *Cb*GAPDH by covalent docking. Overview (A) and active site (B–D) of superimposed apo (green) and holo (cyan) enzymes from different angles.

**Table S1.** Purification table of GAPDH from *Citrobacter* sp. S‐77.
