# Supplementary material for: Glyceraldehyde‐3‐phosphate dehydrogenase from Citrobacter sp. S‐77 is post‐translationally modified by CoA (protein CoAlation) under oxidative stress
Source: FEBS Open Bio. 2018 Nov 28;9(1):53–73. doi: 10.1002/2211-5463.12542 (PMC6325607; doi:10.1002/2211-5463.12542)
Supplement: Supplementary file 1 — Fig. S1. SDS‐PAGE (12.5%) analysis of purified CbGAPDH. Lane 1, low‐molecular‐weight standard marker proteins (MW, 14 400–97 000); lane 2, purified CbGAPDH. [file FEB4-9-53-s001.pdf]

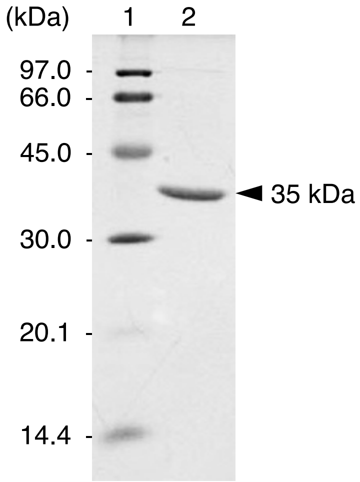

Figure S1. SDS-PAGE (12.5%) analysis of purified *Cb*GAPDH. Lane 1, low-molecular-weight standard marker proteins (MW, 14,400 to 97,000); lane 2, purified *Cb*GAPDH.
