# Supplementary material for: Glyceraldehyde‐3‐phosphate dehydrogenase from Citrobacter sp. S‐77 is post‐translationally modified by CoA (protein CoAlation) under oxidative stress
Source: FEBS Open Bio. 2018 Nov 28;9(1):53–73. doi: 10.1002/2211-5463.12542 (PMC6325607; doi:10.1002/2211-5463.12542)
Supplement: Supplementary file 2 — Fig. S2. Multiple sequence alignment of GAPDH homologs. The sequences of other GAPDHs were extracted from the protein database of NCBI (www.ncbi.nlm.nih.gov); Citrobacter sp. S‐77 GapA (GAN52675.1), Citrobacter sp. S‐77 GapC (WP_045442819), Escherichia coli (P0A9B2), Staphylococcus aureus (Q6GB58), Geobacillus stearothermophilus (P00362), Streptococcus pyrogenes (P0C0G6), Salmonella Typhimurium (P0A1P0), Arabidopsis thaliana (P25856), Homo sapiens (P04406), Oryctolagus cuniculus (P46406) and Rattus norvegicus (P04797). The N‐terminal sequence of CbGAPDH determined in this study is indicated by a red character. The residues conserved in all sequences are shown with a grey background, and the cysteine or conserved histidine residues are shown with yellow or white characters on a black background. [file FEB4-9-53-s002.pdf]

```
C. S-77 GapA             -TIKVGINGFGRIGRIVFRAAQE-RSDIEIVAIND-LLDADYMAYMLKYDSTHGRFNGSV 57
C. S-77 GapC             --SKIGINGFGRIGRLVLRRLLEVKSSVDVVAIND-LTSPKILAYLLKHDSNYGPFPWSV 57
E. coli                  -TIKVGINGFGRIGRIVFRAAQK-RSDIEIVAIND-LLDADYMAYMLKYDSTHGRFDGTV 57
S. aureus                -AVKVAINGFGRIGRLAFRRIQE-VEGLEVVAVND-LTDDDMLAHLLKYDTMQGRFTGEV 57
G. stearothermophilus    -AVKVGINGFGRIGRNVFRAALK-NPDIEVVAVND-LTDANTLAHLLKYDSVHGRLDAEV 57
S. pyrogenes             -VVKVGINGFGRIGRLAFRRIQN-IEGVEVTRIND-LTDPNMLAHLLKYDTTQGRFDGTV 57
S. Typhimurium           -TIKVGINGFGRIGRIVFRAAQK-RSDIEIVAIND-LLDAEYMAYMLKYDSTHGRFDGTV 57
H. sapiens               GKVKVGVNGFGRIGRLVTRAAFN-SGKVDIVAINDPFIDLNYMVYMFQYDSTHGKFHGTV 59
O. cuniculus             --VKVGVNGFGRIGRLVTRAAFN-SGKVDVVAINDPFIDLHYMVYMFQYDSTHGKFHGTV 57
R. norvegicus            --VKVGVNGFGRIGRLVTRAAFS-CDKVDIVAINDPFIDLNYMVYMFQYDSTHGKFNGTV 57

C. S-77 GapA             EVKDGHLIVNGKKIRVTAERDPANLKWDEVGVDVVAEATGLFLTDETARKHITA-GAKKV 116
C. S-77 GapC             DFTEDALIVDGKKIAVYAEKEAKNIPWKTTGAEIVIECTGFYTSTEKANAHLDA-GARKV 116
E. coli                  EVKDGHLIVNGKKIRVTAERDPANLKWDEVGVDVVAEATGLFLTDETARKHITA-GAKKV 116
S. aureus                EVVDGGFRVNGKEVKSFSEPDASKLPWKDLNIDVVLECTGFYTDKDKAQAHIEA-GAKKV 116
G. stearothermophilus    SVNGNNLVVNGKEIIVKAERDPENLAWGEIGVDIVVESTGRFTKREDAAKHLEA-GAKKV 116
S. pyrogenes             EVKEGGFEVNGNFIKVSAERDPENIDWATDGVEIVLEATGFFAKKEAAEKHLHANGAKKV 117
S. Typhimurium           EVKDGHLIVNGKKIRVTAERDPANLKWDEVGVDVVAEATGIFLTDETARKHITA-GAKKV 116
H. sapiens               KAENGKLVINGNPITIFQERDPSKIKWGDAGAEYVVESTGVFTTMEKAGAHLQG-GAKRV 118
O. cuniculus             KAENGKLVINGKAITIFQERDPANIKWGDAGAEYVVESTGVFTTMEKAGAHLKG-GAKRV 116
R. norvegicus            KAENGKLVINGKPITIFQERDPANIKWGDAGAEYVVESTGVFTTMEKAGAHLKG-GAKRV 116

C. S-77 GapA             VLTGPSKDNTPMFVKGANFDKYEGQ--DIVSNASCTTNCLAPLAKVINDNFGIIEGLMTT 174
C. S-77 GapC             LISAPAGD-MKTIVFNVNDDTLDAND-QIISVASCTTNCLAPMAKALHDNFGIQLGTMTT 174
E. coli                  VMTGPSKDNTPMFVKGANFDKYAGQ--DIVSNASCTTNCLAPLAKVINDNFGIIEGLMTT 174
S. aureus                LISAPATGDLKTIVFNTNHQELDGSE-TVVSGASCTTNSLAPVAKVLNDDFGLVEGLMTT 175
G. stearothermophilus    IISAPAKNEDITIVMGVNQDKYDPKAHHVISNASCTTNCLAPFAKVLHEQFGIVRGMMTT 176
S. pyrogenes             VITAPGGNDVKTVVFNTNHDILDGTE-TVISGASCTTNCLAPMAKALHDAFGIQKGLMTT 176
S. Typhimurium           VLTGPSKDNTPMFVKGANFDKYEGQ--DIVSNASCTTNCLAPLAKVINDNFGIIEGLMTT 174
H. sapiens               IISAPSAD-APMFVMGVNHEKYDNSL-KIISNASCTTNCLAPLAKVIHDNFGIVEGLMTT 176
O. cuniculus             IISAPSAD-APMFVMGVNHEKYDNSL-KIVSNASCTTNCLAPLAKVIHDHFGIVEGLMTT 174
R. norvegicus            IISAPSAD-APMFVMGVNHEKYDNSL-KIVSNASCTTNCLAPLAKVIHDNFGIVEGLMTT 174

C. S-77 GapA             VHATTATQKTVDGPS-HKDWRGGRGAAQNIIPSSTGAAKAVGKVLPELNGKLTGMAFRVP 233
C. S-77 GapC             IHAYTGTQSLVDGPR-GKDLRASRAAAENIIPHTTGAAKAIGLVIPALSGKLKGHAQRVP 233
E. coli                  VHATTATQKTVDGPS-HKDWRGGRGASQNIIPSSTGAAKAVGKVLPELNGKLTGMAFRVP 233
S. aureus                IHAYTGDQNTQDAPHRKGDKRRARAAAENIIPNSTGAAKAIGKVIPEIDGKLDGGAQRVP 235
G. stearothermophilus    VHSYTNDQRILDLP--HKDLRRARAAAESIIPTTTGAAKAVALVLPELKGKLNGMAMRVP 234
S. pyrogenes             IHAYTGDQMILDGPHRGGDLRRARAGAANIVPNSTGAAKAIGLVIPELNGKLDGAAQRVP 236
S. Typhimurium           VHATTATQKTVDGPS-HKDWRGGRGASQNIIPSSTGAAKAVGKVLPELNGKLTGMAFRVP 233
H. sapiens               VHAITATQKTVDGPS-GKLWRDGRGALQNIIPASTGAAKAVGKVIPELNGKLTGMAFRVP 235
O. cuniculus             VHAITATQKTVDGPS-GKLWRDGRGAAQNIIPASTGAAKAVGKVIPELNGKLTGMAFRVP 233
R. norvegicus            VHAITATQKTVDGPS-GKLWRDGRGAAQNIIPASTGAAKAVGKVIPELNGKLTGMAFRVP 233

C. S-77 GapA             TPNVSVVDLTVRLEK-AASYEEIKKAIKAASEGPMKGVLGYTEDDVVSTDFNGEVCTSVF 292
C. S-77 GapC             VKTGSVTELVSVLGK-KVTTEDVNNALKKATLNN--ESFGYTDEEIVSSDVIGSHFGSVF 290
E. coli                  TPNVSVVDLTVRLEK-AATYEQIKAAVKAAAEGEMKGVLGYTEDDVVSTDFNGEVCTSVF 292
S. aureus                VATGSLTELTVVLEKQDVTVEQVNEAMKNASNES----FGYTEDEIVSSDVVGMTYGSLF 291
G. stearothermophilus    TPNVSVVDLVAELEK-EVTVEEVNAALKAAAEGELKGILAYSEEPLVSRDYNGSTVSSTI 293
S. pyrogenes             VPTGSVTELVVTLDK-NVSVDEINSAMKAASNDS----FGYTEDPIVSSDIVGVSYGSLF 291
S. Typhimurium           TPNVSVVDLTVRLEK-AATYEQIKAAVKAAAEGEMKGVLGYTEDDVVSTDFNGEVCTSVF 292
H. sapiens               TANVSVVDLTCRLEK-PAKYDDIKKVVKQASEGPLKGILGYTEHQVVSSDFNSDTHSSTF 294
O. cuniculus             TPNVSVVDLTCRLEK-AAKYDDIKKVVKQASEGPLKGILGYTEDQVVSCDFNSATHSSTF 292
R. norvegicus            TPNVSVVDLTCRLEK-PAKYDDIKKVVKQAAEGPLKGILGYTEDQVVSCDFNSNSHSSTF 292

C. S-77 GapA             DAKAGIALN---DNFVKLVSWYDNETGYSNKVLDLIAHISK--- 330
C. S-77 GapC             DATQTEITEVGDLQLVKTVAWYDNEYGFVTQLVRTLEKLIKL-- 332
E. coli                  DAKAGIALN---DNFVKLVSWYDNETGYSNKVLDLIAHISK--- 330
S. aureus                DATQTRVMSVGDRQLVKVAAWYDNEMSYTAQLVRTLAYLAELSK 335
G. stearothermophilus    DALSTMVID---GKMVKVVSWYDNETGYSHRVVDLAAYIASKGL 334
S. pyrogenes             DATQTKVMEVDGSQLVKVVSWYDNEMSYTAQLVRTLEYFAKIAK 335
S. Typhimurium           DAKAGIALN---DNFVKLVSWYDNETGYSNKVLDLIAHISK--- 330
H. sapiens               DAGAGIALN---DHFVKLISWYDNEFGYSNRVVDLMAHMASKE- 334
O. cuniculus             DAGAGIALN---DHFVKLISWYDNEFGYSNRVVDLMVHMASKE- 332
R. norvegicus            DAGAGIALN---DNFVKLISWYDNEYGYSNRVVDLMAYMASKE- 332
```

Fig. S2

Figure S2. Multiple sequence alignment of GAPDH homologs. The sequences of other GAPDHs were extracted from the protein database of NCBI (www.ncbi.nlm.nih.gov); *Citrobacter* sp. S-77 GapA (GAN52675.1), *Citrobacter* sp. S-77 GapC (WP_045442819), *Escherichia coli* (P0A9B2), *Staphylococcus aureus* (Q6GB58), *Geobacillus stearothermophilus* (P00362), *Streptococcus pyrogenes* (P0C0G6), *Salmonella* Typhimurium (P0A1P0), *Arabidopsis thaliana* (P25856), *Homo sapiens* (P04406), *Oryctolagus cuniculus* (P46406) and *Rattus norvegicus* (P04797). The N-terminal sequence of *Cb*GAPDH determined in this study is indicated by a red character. The residues conserved in all sequences are shown with a grey background, and the cysteine or conserved histidine residues are shown with yellow or white characters on a black background.
