# Supplementary material for: Glyceraldehyde‐3‐phosphate dehydrogenase from Citrobacter sp. S‐77 is post‐translationally modified by CoA (protein CoAlation) under oxidative stress
Source: FEBS Open Bio. 2018 Nov 28;9(1):53–73. doi: 10.1002/2211-5463.12542 (PMC6325607; doi:10.1002/2211-5463.12542)
Supplement: Supplementary file 3 — Fig. S3. Peptide mass fingerprinting of native and CoAlated CbGAPDH in vitro. The mass spectra of native CbGAPDH (A) and CAM treated CbGAPDH (B) showed that the mass shift of 2CAM (114 Da) in Cys149 and 153 containing peptide, and 1CAM (57 Da) in Cys288 containing peptide. These results indicated that in our experimental conditions, incubation of the enzyme with 10 mm IAM for 60 min in dark was enough to totally proceed carbamidomethylation for alkylation of free cysteines in CbGAPDH. (C) In vitro CoAlation of CbGAPDH occurred in Cys149 and 153 containing peptide, while Cys288 was exclusively carbamidomethylated. Intramolecular disulphide bonding (Cys149‐S‐S‐Cys153) was also detected as an alternative redox regulatory mechanism. (D) These redox modifications were reversed in a DTT dependent manner since CAM modified Cys149 and 153 containing peptide was detected. Note that the spectrum of (C) was recorded with linear negative mode, while the other spectra were recorded in linear positive mode. [file FEB4-9-53-s003.pdf]

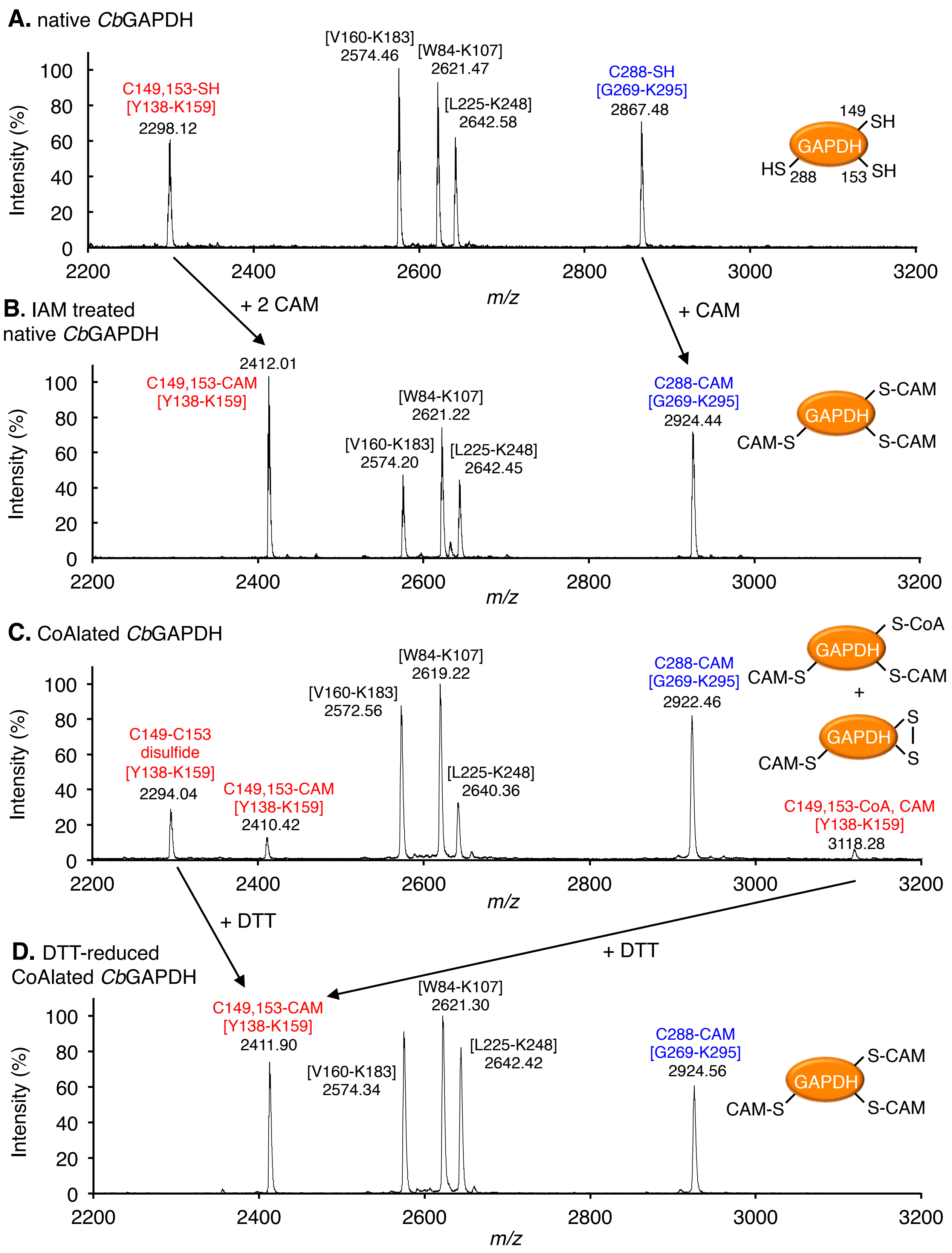

A. native CbGAPDH
Intensity (%)
m/z
C149,153-SH
[Y138-K159]
2298.12
[V160-K183]
2574.46
[W84-K107]
2621.47
[L225-K248]
2642.58
C288-SH
[G269-K295]
2867.48
GAPDH
149 SH
HS 288
153 SH
+ 2 CAM
+ CAM
B. IAM treated native CbGAPDH
2412.01
C149,153-CAM
[Y138-K159]
[W84-K107]
2621.22
[V160-K183]
2574.20
[L225-K248]
2642.45
C288-CAM
[G269-K295]
2924.44
S-CAM
CAM-S
S-CAM
C. CoAlated CbGAPDH
[W84-K107]
2619.22
[V160-K183]
2572.56
C288-CAM
[G269-K295]
2922.46
S-CoA
CAM-S
S-CAM
+
S
S
CAM-S
C149-C153
disulfide
[Y138-K159]
2294.04
C149,153-CAM
[Y138-K159]
2410.42
[L225-K248]
2640.36
C149,153-CoA, CAM
[Y138-K159]
3118.28
+ DTT
+ DTT
D. DTT-reduced CoAlated CbGAPDH
C149,153-CAM
[Y138-K159]
2411.90
[W84-K107]
2621.30
[L225-K248]
2642.42
[V160-K183]
2574.34
C288-CAM
[G269-K295]
2924.56
S-CAM
CAM-S
S-CAM

Fig. S3

Fig. S3. Peptide mass fingerprinting of native and CoAlated *Cb*GAPDH i*n vitro*. The mass spectra of native *Cb*GAPDH (A) and CAM treated *Cb*GAPDH (B) showed that the mass shift of 2CAM (114 Da) in Cys149 and 153 containing peptide, and 1CAM (57 Da) in Cys288 containing peptide. These results indicated that in our experimental conditions, incubation of the enzyme with 10 mM IAM for 60 min in dark was enough to totally proceed carbamidomethylation for alkylation of free cysteines in *Cb*GAPDH. (C) *In vitro* CoAlation of *Cb*GAPDH occurred in Cys149 and 153 containing peptide, while Cys288 was exclusively carbamidomethylated. Intramolecular disulphide bonding (Cys149-S-S-Cys153) was also detected as an alternative redox regulatory mechanism. (D) These redox modifications were reversed in a DTT dependent manner since CAM modified Cys149 and 153 containing peptide was detected. Note that the spectrum of (C) was recorded with linear negative mode, while the other spectra were recorded in linear positive mode.
