# Supplementary material for: Glyceraldehyde‐3‐phosphate dehydrogenase from Citrobacter sp. S‐77 is post‐translationally modified by CoA (protein CoAlation) under oxidative stress
Source: FEBS Open Bio. 2018 Nov 28;9(1):53–73. doi: 10.1002/2211-5463.12542 (PMC6325607; doi:10.1002/2211-5463.12542)
Supplement: Supplementary file 4 — Fig. S4. MS/MS spectrum of in vitro CoAlated CbGAPDH. Peptide with Cys288 remains carbamidomethylated after our CoAlation assay condition. [file FEB4-9-53-s004.pdf]

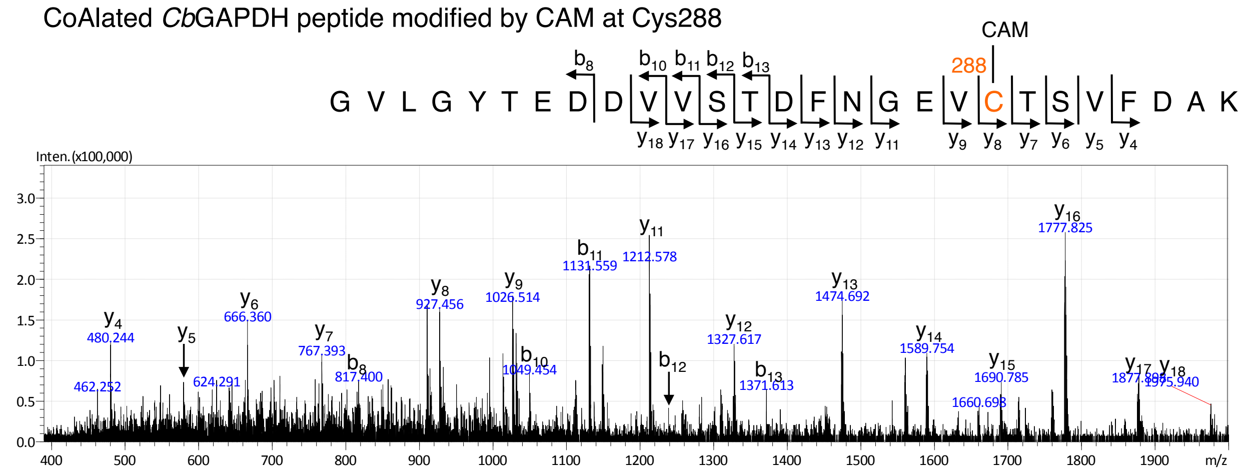

Figure S4. MS/MS spectrum of *in vitro* CoAlated *Cb*GAPDH. Peptide with Cys288 remains carbamidomethylated after our CoAlation assay condition.
