# Supplementary material for: Glyceraldehyde‐3‐phosphate dehydrogenase from Citrobacter sp. S‐77 is post‐translationally modified by CoA (protein CoAlation) under oxidative stress
Source: FEBS Open Bio. 2018 Nov 28;9(1):53–73. doi: 10.1002/2211-5463.12542 (PMC6325607; doi:10.1002/2211-5463.12542)
Supplement: Supplementary file 5 — Fig. S5. MS/MS spectra of native CbGAPDH. (A) Carbamidomethylated peptide at Cys149 and 153. (B) Carbamidomethylated peptide at Cys288. [file FEB4-9-53-s005.pdf]

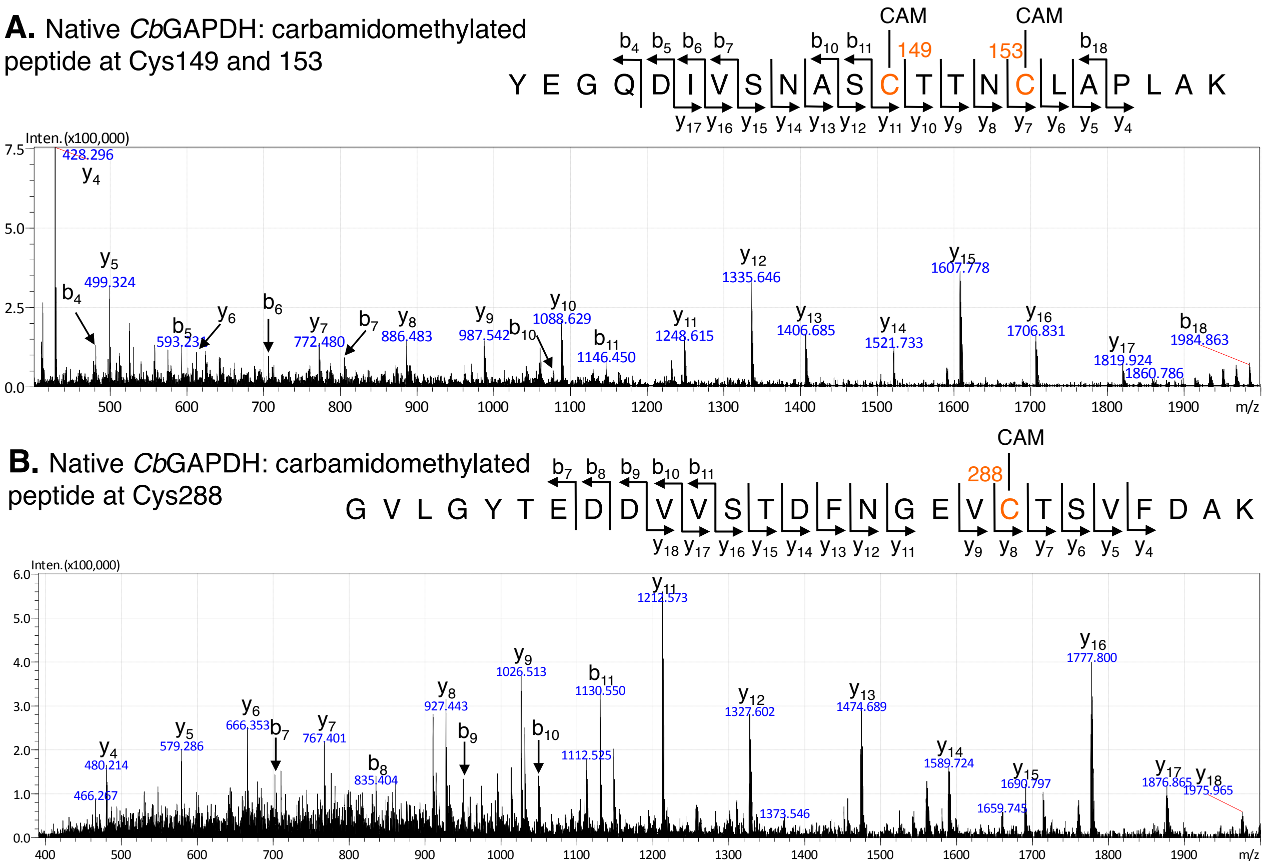

Figure S5. MS/MS spectra of native *Cb*GAPDH. (A) Carbamidomethylated peptide at Cys149 and 153. (B) Carbamidomethylated peptide at Cys288.
