# Supplementary material for: Glyceraldehyde‐3‐phosphate dehydrogenase from Citrobacter sp. S‐77 is post‐translationally modified by CoA (protein CoAlation) under oxidative stress
Source: FEBS Open Bio. 2018 Nov 28;9(1):53–73. doi: 10.1002/2211-5463.12542 (PMC6325607; doi:10.1002/2211-5463.12542)
Supplement: Supplementary file 6 — Fig. S6. MS/MS spectra of in vitro overoxidised CbGAPDH by 1 mm H2O2. (A) Dimedone trapped indicated the formation of labile intermediate sulphenate at Cys149, while Cys153 is carbamidomethylated. (B) Over‐oxidation of Cys149 was confirmed, while Cys153 is carbamidomethylated. (C) Cys149‐S‐S‐Cys153 intramolecular disulphide bonding was also detected. [file FEB4-9-53-s006.pdf]

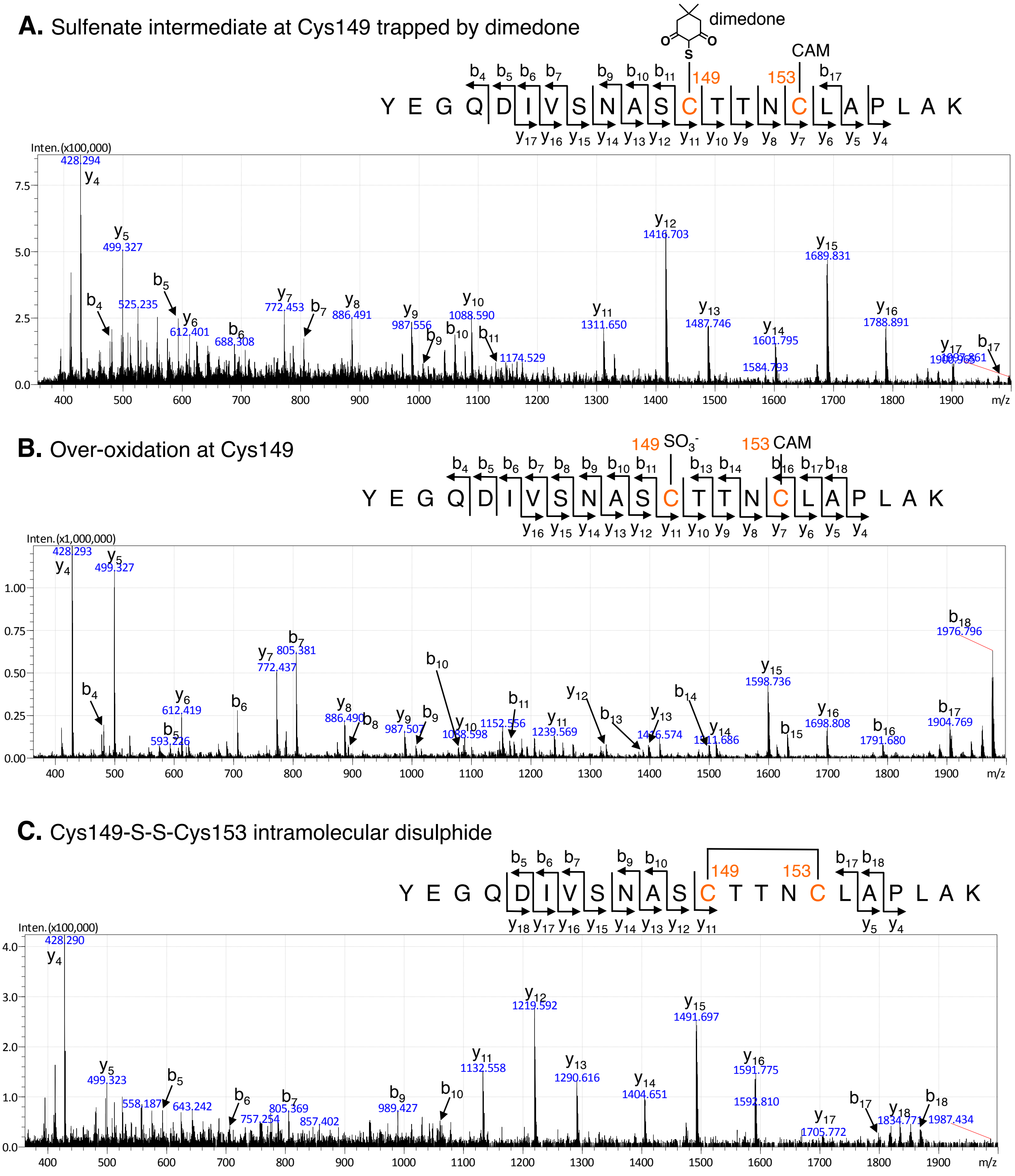

Figure S6. MS/MS spectra of over-oxidised *Cb*GAPDH by 1 mM H_2O_2. (A) Dimedone trapped indicated the formation of labile intermediate sulphenate at Cys149, while Cys153 is carbamidomethylated. (B) Over-oxidation of Cys149 was confirmed, while Cys153 is carbamidomethylated. (C) Cys149-S-S-Cys153 intramolecular disulphide bonding was also detected.
