# Supplementary material for: Glyceraldehyde‐3‐phosphate dehydrogenase from Citrobacter sp. S‐77 is post‐translationally modified by CoA (protein CoAlation) under oxidative stress
Source: FEBS Open Bio. 2018 Nov 28;9(1):53–73. doi: 10.1002/2211-5463.12542 (PMC6325607; doi:10.1002/2211-5463.12542)
Supplement: Supplementary file 7 — Fig. S7. Inactivation of CbGAPDH by NaOCl with/without CoA and the reversibility by DTT in vitro. CbGAPDH was incubated at each indicated conditions in the buffer solution of 100 mm Tris/HCl (pH 7.5) at 25 °C for 15 min and subsequently treated with 10 mm DTT for 15 min. The residual activity was then determined before (black bar) and after (white bar) the treatment of DTT. The control experiment (without any treatment) is given for comparison. Activities are given as a percentage of the initial activity (100 ± 5.3 U·mg−1) before the inactivation experiment. The results are presented as means of at least three independent experiments with standard deviation. [file FEB4-9-53-s007.pdf]

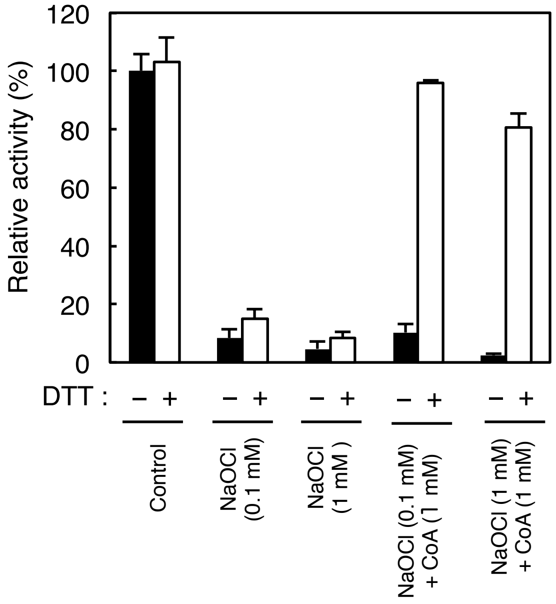

Figure S7. Inactivation of *Cb*GAPDH by NaOCl with/without CoA and the reversibility by DTT *in vitro*. *Cb*GAPDH was incubated at each indicated conditions in the buffer solution of 100 mM Tris/HCl (pH 7.5) at 25 °C for 15 min and subsequently treated with 10 mM DTT for 15 min. The residual activity was then determined before (black bar) and after (white bar) the treatment of DTT. The control experiment (without any treatment) is given for comparison. Activities are given as a percentage of the initial activity (100 ± 5.3 U/mg) before the inactivation experiment. The results are presented as means of at least three independent experiments with standard deviation.
