# Supplementary material for: Glyceraldehyde‐3‐phosphate dehydrogenase from Citrobacter sp. S‐77 is post‐translationally modified by CoA (protein CoAlation) under oxidative stress
Source: FEBS Open Bio. 2018 Nov 28;9(1):53–73. doi: 10.1002/2211-5463.12542 (PMC6325607; doi:10.1002/2211-5463.12542)
Supplement: Supplementary file 8 — Fig. S8. MALDI‐TOF mass spectra of CbGAPDH treated with NaOCl plus CoA in vitro. The mass spectra of CbGAPDH incubated with 0.1 mm NaOCl in the presence of 1 mm CoA (30 min) were acquired before (A) and after (B) the treatment with 10 mm DTT (30 min). The peaks marked by * is assigned to the adducts of sinapinic acid. [file FEB4-9-53-s008.pdf]

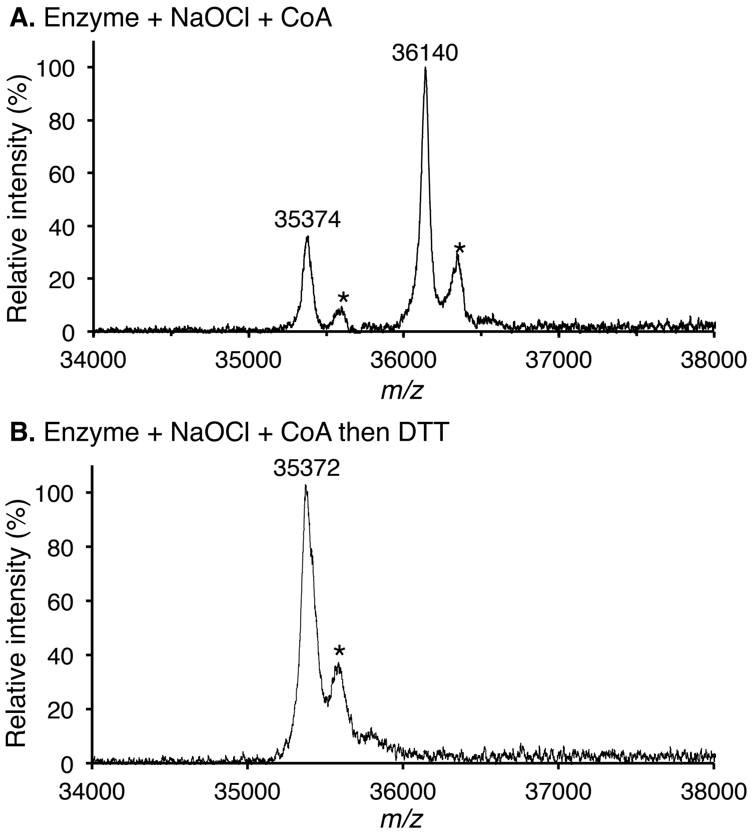

Figure S8. MALDI-TOF mass spectra of *Cb*GAPDH treated with NaOCl plus CoA. The mass spectra of *Cb*GAPDH incubated with 0.1 mM NaOCl in the presence of 1 mM CoA (30 min) were acquired before (A) and after (B) the treatment with 10 mM DTT (30 min). The peaks marked by * is assigned to the adducts of sinapinic acid.
