# Supplementary material for: Glyceraldehyde‐3‐phosphate dehydrogenase from Citrobacter sp. S‐77 is post‐translationally modified by CoA (protein CoAlation) under oxidative stress
Source: FEBS Open Bio. 2018 Nov 28;9(1):53–73. doi: 10.1002/2211-5463.12542 (PMC6325607; doi:10.1002/2211-5463.12542)
Supplement: Supplementary file 9 — Fig. S9. MS/MS spectra of overoxidised CbGAPDH by 1 mm NaOCl in vitro. (A) Over‐oxidation of Cys149 was confirmed, while Cys153 is carbamidomethylated. (B) Cys149‐S‐S‐Cys153 intramolecular disulphide bonding was also detected. [file FEB4-9-53-s009.pdf]

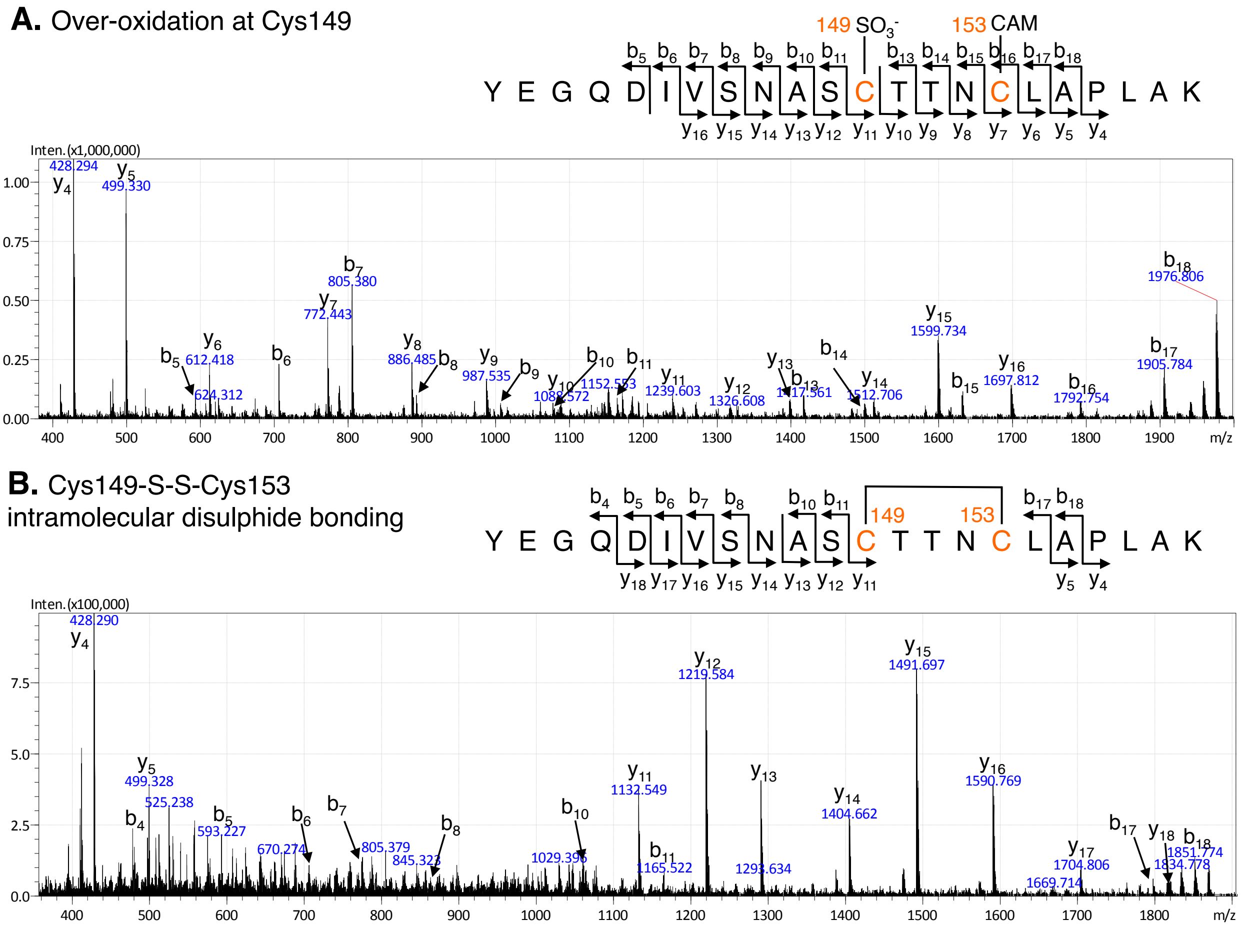

Figure S9. MS/MS spectra of over-oxidised *Cb*GAPDH by 1 mM NaOCl. (A) Over-oxidation of Cys149 was confirmed, while Cys153 is carbamidomethylated. (B) Cys149-S-S-Cys153 intramolecular disulphide bonding was also detected.
