# Supplementary material for: Glyceraldehyde‐3‐phosphate dehydrogenase from Citrobacter sp. S‐77 is post‐translationally modified by CoA (protein CoAlation) under oxidative stress
Source: FEBS Open Bio. 2018 Nov 28;9(1):53–73. doi: 10.1002/2211-5463.12542 (PMC6325607; doi:10.1002/2211-5463.12542)
Supplement: Supplementary file 10 — Fig. S10. Far‐UV CD spectra of native (black line) and CoAlated (red dotted line) CbGAPDH. The spectra were recorded in 190–260 nm, and the data are presented as molar ellipticity. Each spectra line was plotted by the average of three scans. [file FEB4-9-53-s010.pdf]

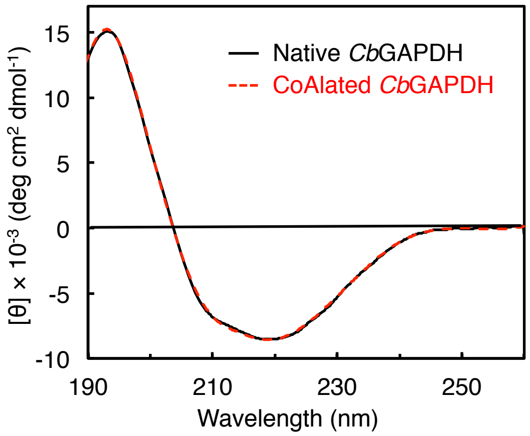

Figure S10. Far-UV CD spectra of native (black line) and CoAlated (red dotted line) *Cb*GAPDH. The spectra were recorded in 190-260 nm, and the data are presented as molar ellipticity.Each spectra line was plotted by the average of three scans.
