# Supplementary material for: Glyceraldehyde‐3‐phosphate dehydrogenase from Citrobacter sp. S‐77 is post‐translationally modified by CoA (protein CoAlation) under oxidative stress
Source: FEBS Open Bio. 2018 Nov 28;9(1):53–73. doi: 10.1002/2211-5463.12542 (PMC6325607; doi:10.1002/2211-5463.12542)
Supplement: Supplementary file 11 — Fig. S11. Modelled structures of CoAlated CbGAPDH by covalent docking. Overview (A) and active site (B–D) of superimposed apo (green) and holo (cyan) enzymes from different angles. [file FEB4-9-53-s011.pdf]

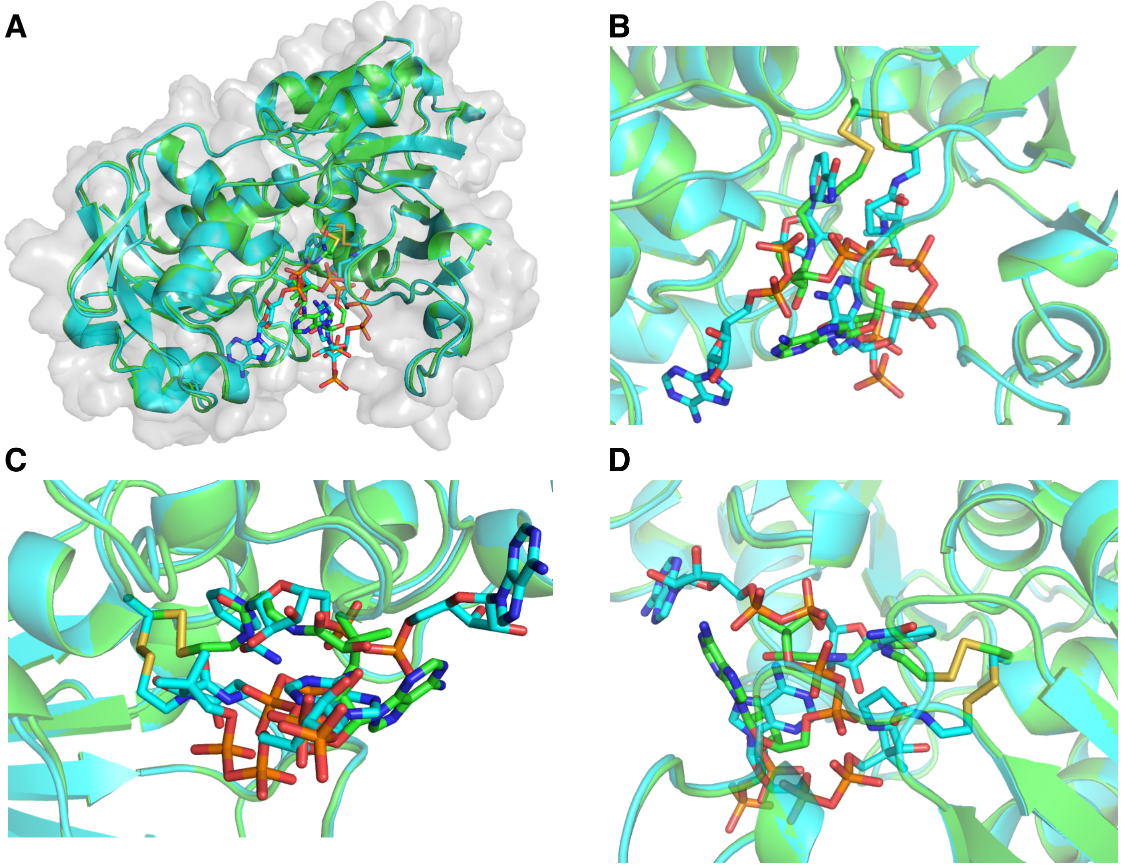

Figure S11. Modelled structures of CoAlated *Cb*GAPDH by covalent docking. Overview (A) and active site (B, C, D) of superimposed apo (green) and holo (cyan) enzymes from different angles
