# Supplementary material for: Glyceraldehyde‐3‐phosphate dehydrogenase from Citrobacter sp. S‐77 is post‐translationally modified by CoA (protein CoAlation) under oxidative stress
Source: FEBS Open Bio. 2018 Nov 28;9(1):53–73. doi: 10.1002/2211-5463.12542 (PMC6325607; doi:10.1002/2211-5463.12542)
Supplement: Supplementary file 12 — Table S1. Purification table of GAPDH from Citrobacter sp. S‐77. [file FEB4-9-53-s012.docx]

**Table S1.** Purification table of GAPDH from *Citrobacter* sp. S-77

| Step | Total activity | Total protein | Specific activity | Purification | Yield |
| --- | --- | --- | --- | --- | --- |
|  | (Units) | (mg) | (Units/mg) | fold | % |
| Soluble fraction | 6635 | 225 | 29.4 | 1 | 100 |
| Hydroxyapatite | 4113 | 43 | 96.0 | 3.3 | 62 |
| Superdex200 | 1456 | 10 | 145.6 | 4.9 | 22 |

The enzyme assays were performed in 100 mM Tris/HCl (pH 8.0) with 1 mM DTT, 40 mM KH_2_PO_4_, 1 mM NAD^+^ and 6 mM glyceraldehyde-3-phosphate (G3P) at 30 °C.
